# Peculiar Structural Effects in Pure and Doped Functional Single Crystals of Complex Compositions

**DOI:** 10.3390/molecules25102451

**Published:** 2020-05-25

**Authors:** Galina Kuz’micheva, Irina Kaurova

**Affiliations:** MIREA-Russian Technological University, Vernadskogo pr. 78, Moscow 119454, Russia; galina_kuzmicheva@list.ru

**Keywords:** functional materials, crystal growth, doping, solid solution, X-ray diffraction (XRD), neutron diffraction, X-ray absorption spectroscopy, crystal structure, local structure, symmetry

## Abstract

Results of a detailed structural characterization of nominally pure and doped single crystals of scheelite, eulytin, and perovskite families obtained by melt methods were considered and analyzed. The influence of growth and post-growth annealing conditions on actual compositions of crystals is shown. The reasons for the coloration of the crystals are explained. A change in crystal symmetry due to crystal–chemical and growth reasons is considered. The use of structural analysis and X-ray absorption spectroscopy is substantiated to reveal the role of activator ions in the formation of statistical and local structures, respectively. A relationship between the distribution of activator ions over crystallographic sites and photoluminescent parameters of materials is established, which allows selecting optimal systems for the application. The combined results of studying single-crystal compounds of other classes (huntite, sillenite, whitlockite, garnet, tetragonal bronzes) allow formulating and summarizing structural effects that appeared in the systems and caused by various factors and, in many cases, due to the local environment of cations. A principal difference in the structural behavior of solid solutions and doped compounds is shown. The methodology developed for single-crystal samples of complex compositions can be recommended for the systematic structural studies of functional materials of different compositions.

## 1. Introduction

Fundamental conditions that determine the competitiveness of crystalline multifunctional media and the possibility of their practical use are technological effectiveness and high operational characteristics that can be controlled. This can be achieved either by the development of compounds with fundamentally new compositions and structures or by the optimization of known systems with a formation of new functional properties. The last way is preferable due to its cost-effectiveness and possibility of varying properties of the material, which has already shown its practical significance and has the proven growth technology.

A simple technological technique to achieve and control the required functional characteristics of the material is its activation by impurities of different nature, type, concentration, and method of introduction. Activation is a doping of a system with small amount of active impurity ions (~<5 wt%) that can be described by quasi-chemical equations involving all the main types of point defects in concentrations comparable to each other (intrinsic point defects: vacancies, self-interstitial atoms, antisite defects; extrinsic point defects: substitutional or interstitial foreign atoms). Activation is the most effective process for modifying compounds, which allows obtaining effects of an incomparable level by an introduction of small amounts of activator ions into a crystal matrix [1,2]. In most cases, dopants are introduced over stoichiometry, which leads to difficulties in studying such objects [3].

Solid solutions differ significantly from compounds doped with activator ions, primarily, in a higher content of components added to a system (~>5 wt%) [3]. The system, which is planned to be observed in the finished product, i.e., substitutional and/or interstitial solid solution, is already created at the charge level of the growth process. In other words, a formation of desired solid solutions (first of all, substitutional one) is carried out already at the stage of selecting initial components and their ratio based on the theory of isomorphic substitution [4].

It should be noted that commercial bulk single crystals, obtained, as a rule, by the most technologically advanced melt methods (crystals of the scheelite, perovskite, whitlockite, huntite, garnet, and other families), can have a uniform crystal composition only for compounds with congruent melting (CM) [5,6]. However, even in the case of CM, the composition of the grown crystal (actual composition) in most cases differs from the composition of the initial charge due to growth defects (the facet effect, growth striations, etc.) and activator ions introduced into a system [7,8]. The same defects are also observed in crystals obtained from solution, for example, by the hydrothermal method (KH_2_PO_4_ crystals) [9,10,11]. An investigation of structural features and actual compositions of crystals, taking into account all types and concentrations of point defects, is one of the urgent tasks of modern science, since defects that form the actual crystal structure have a significant impact on the operational properties of the material [7]. Therefore, knowledge of the structural characteristics of a compound is necessary for the growth of functional materials with variable parameters.

The main sources of structural information are diffraction methods that provide the most complete and comprehensive information on the structure of crystalline compounds. However, the sensitivity of these methods decreases sharply for disordered structures, which can lead to errors in the determination of structural parameters, for example, interatomic distances, reaching several tenths of an angstrom. Similar problems arise during diffraction studies of defective structures or structures having different atoms located in the same crystallographic site. In this case, due to the strong correlation of parameters that occurs during the structure refinement process, actual structural parameters can differ significantly from average values obtained based on diffraction data. In the X-ray diffraction experiment, additional difficulties occur, in particular, low accuracy of determining the positions of light atoms against a background of heavy atoms, difficult separation of atoms with close form factors (close atomic numbers), and difficult determination of the content of oxygen vacancies.

In this regard, a high-priority objective is an investigation of structures of compounds using a combination of several methods and techniques. One of the promising research methods that proved its effectiveness in applying to a wide range of tasks is X-ray absorption spectroscopy (XAS), which allows obtaining structural information about the local structure in low-order systems [12,13]. XAS allows determining interatomic distances with high accuracy and, in addition, this method is highly sensitive to local distortions in highly symmetric structures. An XAS investigation of compounds of complex compositions is justified since it allows obtaining specified information on the position of atoms, their relative displacements, changes in the local atomic structure resulted from activation or formation of solid solutions [12,13]. Operational parameters of functional materials strongly depend on crystal structures and compositions, which, in turn, are determined by the methods (conditions) of synthesis and/or processing. A determination of correlations between physical properties and crystal structure (statistical and/or local) is a fundamental task, which is still very important and relevant despite decades of intensive research.

The purpose of the study is to establish a combined influence of growth conditions, compositions of solid solutions and nature and concentration of activator ions on the fundamental (composition and structure) and functional (in particular, optical) properties of bulk single crystals of complex compositions.

## 2. Functional Crystals of Complex Compositions: General Information

The objects of study are compounds of well-known families that either have already been applied in practice or demonstrate a set of promising properties. The review considers both nominally pure and doped compounds of the following families: perovskite, CsCd*X*_3_ (*X* = Cl, Br) and TlCd*X*_3_ (*X* = Cl, I) compounds; scheelite, SrMoO_4_, Pb*T*O_4_ and (Na,*RE*)*T*O_4_ (*RE*^3+^ = La, Gd; *T* = Mo, W); eulytin, Bi_4_Ge_3_O_12_. Other families of compounds or individual compounds, for which the problems considered in this review are relevant, are also involved.

On one hand, an increased interest in such materials is due, to their high scientific importance for solving such fundamental problems as determination of correlations between their composition, structure and properties and development the theory of isomorphic miscibility of components. On the other hand, these compounds have widespread practical use in various fields of electronics, in particular, optoelectronics, laser optics, acousto-optics, etc. It should be noted that structures of the above-mentioned compounds have been intensively and successfully studied for several decades. However, some features of their structures are still the subject of intensive scientific discussions and remain one of the urgent problems of fundamental and applied materials science. Moreover, in the vast majority of cases, publications concern the compositions of initial charges rather than the actual refined compositions of grown crystals. As a result, operational properties and other characteristics of crystals are attributed to the initial charge composition, which is fundamentally wrong.

### 2.1. Scheelite Family Compounds

Powellite CaMoO_4_ [14], wulfenite PbMoO_4_ [15], stolzite PbWO_4_ [15], SrMoO_4_ [16] and phases with the general composition (Na^1+^_0.5_*RE*_0.5_)*T*O_4_ with the *RE*^3+^ = La, Gd and *T* = Mo, W [17] crystallizes in the scheelite structure.

Molybdates and tungstates with the scheelite structure exhibit, first of all, promising optical and spectral-luminescent properties. They are widely used as laser materials, phosphors, and optical functional media [18,19,20] as well as scintillation (cryogenic) detectors for rare-event and dark matter searches [21,22,23]. Scheelite-type molybdates, in particular CaMoO_4_ and PbMoO_4_, possess high acousto-optical characteristics and are actively used as optical solid-state deflectors, modulators, adjustable filters, and surface acoustic wave devices [24,25]. In turn, single crystals of double rare-earth sodium tungstates and molybdates are of considerable interest as nonlinear elements for laser Raman shifting and active media for solid-state, tunable, thin-disk and ultrashort (<100 fs) pulsed lasers [20,25].

In recent years, molybdates and tungstates with scheelite structure are increasingly used in modern technology due to the possible variation of their physicochemical, electrophysical, and optical characteristics in a wide range of compositions. First of all, this becomes possible due to doping with rare-earth metal ions (*RE^3+^*). These ions substitute for divalent metal ions without significant distortion of the crystal structure (as stated in literature without any experimental evidence base).

#### 2.1.1. PbTO_4_ (T = Mo,W), Pb(Mo,W)O_4_, PbMoO_4_:Nd^3+^

Lead molybdate PbMoO_4_ (PMO) and lead tungstate PbWO_4_ (PWO) are multifunctional inorganic semiconductors with luminescence and stimulated Raman scatting behavior. They are actively used as optical and acousto-optical media, phosphors [26,27] and cryogenic phonon scintillation detectors to study neutrinoless double beta decay in^180,186^ W and ^92,98,100^Mo [23,26]. These materials are also used as a host, in particular for lanthanide activators (Nd^3+^, Eu^3+^, Tb^3+,^ etc.). In PMO and PWO crystals doped with the Nd^3+^ ions, a simultaneous laser generation and SRS conversion (SRS, stimulated Raman scattering) were obtained under different types and conditions of pumping [19]. It should be noted that PbMoO_4_ and PbWO_4_ form solid solutions over all the regions of compositions [28]. PbMo*_x_*W_1−*x*_O_4_ crystals combine good mechanical properties of PbMoO_4_ with the better optical transmission in the short wavelength range of PbWO_4_ [29]. At the same time in the PbMo*_x_*W_1−*x*_O_4_ crystal, the multi-Stokes SRS oscillations were observed containing different combinations of the Raman frequency shifts corresponding to a structure of the plumbic molybdate and tungstate [29].

Active use of PMO and PWO crystals is limited by the presence of additional optical absorption. According to Bochkova et al. [30], the absorption in the PMO crystal is caused by anti-structural defects Mo_Pb_^n•^ (lead atoms in the molybdenum site) or by the cation recharge Mo^6+^ + Pb^2+^ ↔ Mo^5+^ + Pb^3+^. Moreover, a heterovalent activation occurs in PMO and PWO crystals doped with *RE*^3+^ laser ions, which should lead to the formation of additional types of point defects that are absent in nominally pure crystals. Impedance spectroscopic investigations of Nd^3+^ [31] and La^3+^-doped [32] PWO crystals showed that for low doping concentrations (up to 1 mol%), Nd^3+^ or La^3+^ ions substitute for the Pb^2+^ ones. In this case, charge compensation occurs due to the formation of vacancies in the Pb site. For higher doping concentrations (more than 1.5 wt% [33] and 1 mol% [32]), Nd^3+^ is incorporated into the Pb^2+^ and W^6+^ sublattices, the excessive negative charge being compensated by oxygen vacancies [33]; La^3+^ ions enter the W^6+^ sublattice and charge compensation is due to the interstitial oxygen [32]. The occurrence of La ions in the W lattice and existence of interstitial oxygen in 1.0- and 2.5-mol% PWO:La crystal samples is confirmed by the results of X-ray photoelectron spectroscopy and extended X-ray absorption fine spectroscopy measurements of W–L_3_ absorption [34]. In addition to the concentration of dopant ions, a formation of point defects is also affected by the doping schemes, in particular, the initial composition of the compound containing dopant ions [33]. Refinement of PMO and PWO crystal structures were performed based on a single-crystal X-ray experiment [35] and by the Rietveld method [36], however, the occupancy factors for the crystallographic sites were not refined and site deficiency was not estimated.

PMO and PWO single crystals are usually grown by the Czochralski method [26,33], as well as by the solvothermal [37] and hydrothermal [27] processes, Bridgman method [38] and flux growth technique [39]. When single crystals are grown by the Czochralski method, the crystal stoichiometry is violated due to the higher vapor pressure of MoO_3_/WO_3_ compared to PbO. As a result, crystal growth occurs from the MoO_3_/WO_3_ enriched melt [33], which can induce an appearance of structural defects of various types.

#### 2.1.2. SrMoO_4_ and SrMoO_4_:RE^3+^

Strontium molybdate SrMoO_4_ (SMO) is a wide-gap semiconductor and efficient luminescent material applied in the phosphor industry to create solid-state light-emitting diodes (LED) including white LEDs [40]. This multifunctional material is also used as eye-safe Raman lasers [41], sensors [42], photocatalysts [43], scintillation detectors [44], electro-optic and acousto-optical devices [45].

The functional properties of phosphors are influenced by the synthesis conditions, especially by the temperature regime [46], which affects the actual crystal composition. The unit cell parameters (structural parameters) and the band gap are indicators of composition and vary depending on the deficiency of crystal structure. However, the number of works focused on a systematic study of the crystal structure, electronic structure and optical properties of doped SMO crystals is extremely limited.

Since the *RE*^3+^ activators (extrinsic point defects) have formal charges different from that of the Sr^2+^ in SMO, their introduction into the matrix should inevitably lead to the appearance of additional point defects to preserve the electroneutrality in the system. However, comprehensive structural studies of SrMoO_4_:*RE*^3+^ crystals with a determination of their actual compositions are almost absent in the literature. Only several works contain any experimental evidence of the location of dopant ions in the SMO crystal structure. Using the full-profile Rietveld method, Park et al. [47] established that the replacement of Sr^2+^ ions with Tb^3+^ ones leads to the appearance of vacancies in all crystallographic sites of the structure: (Sr_0.897−0.944_Tb_0.029−0.038_⎕_0.018−0.074_)(Mo_0.933−0.986_⎕_0.014−0.067_)(O_0.851−1.000_⎕_0.000−0.149_) (vacancies are marked with a square, ⎕). Based on the Rietveld refinement, Shivakumara et al. [40] found that the smaller ionic radii of 8-coordinated Eu^3+^ ion (r_Eu_^VIII^ = 1.066 Å) are incorporated into the larger ionic radii of 8-coordinated Sr^2+^ ion in the SMO structure. Lin et al. [48] stated that the Sm^3+^ ions enter the SMO host crystal lattice and preferentially substitute for Sr^2+^ ions that lead to charge unbalance, which can be eliminated by introducing alkali metal ions (Li^+^, Na^+^, K^+^) entered the same Sr site. Electron paramagnetic resonance (EPR) spectroscopy of electronic states and local structures for Er^3+^ in SMO showed that the Sr^2+^ site is occupied by the Er^3+^ impurity [49]. Based on the X-ray diffraction data of SrMoO_4_:Tb^3+^ nanocrystals, it was concluded that Sr^2+^ ions are partially replaced by Tb^3+^ ions, but the mechanisms of charge compensation were not discussed [50]. Partial replacement of Sr^2+^ ions in SMO by rare-earth ions was supposed in Refs [51,52,53].

Single-crystal SMO, both nominally pure and doped with luminescent ions, are generally grown by the Czochralski method [51,52,53,54] as well as by the modified Stepanov technique [55] and the flux-growth technique [56]. An introduction of dopants in the form of RE^3+^NbO_4_ into the SrMoO_4_ melt ensures the growth of electroneutral crystals without any formation of intrinsic charged defects since the replacement of Sr^2+^ ions with *RE*^3+^ ions is compensated by Nb^5+^ ions entering the Mo^6+^ sites [53,55]. However, this is not confirmed by any structural studies.

#### 2.1.3. (Na,RE)TO_4_ (T = Mo, W; RE^3+^ = La,Gd)

Sodium-containing double molybdates and tungstates with the general chemical formula (Na^1+^_0.5_*RE*^3+^_0.5_)*T*O_4_ (*RE*^3+^ is a rare-earth element; *T* = Mo, W), both nominally pure and doped with rare-earth ions, are optical materials used in diode-pumped tunable solid-state lasers and ultrashort pulse lasers [57]. Activation of this material with rare-earth ions allows its use as phosphors [58].

X-ray diffraction study of nominally pure and 5-mol% Yb^3+^-doped (Na,Gd)WO_4_ crystals showed their crystallization in the space group *I*4_1_/*a* without any refinement of actual composition [59,60]. At the same time, Cascales et al. [61] performed an X-ray diffraction investigation of (Na,Gd)WO_4_ single crystals, both undoped and doped with up to 20-mol% Yb^3+^, and established the noncentrosymmetric space group $I\bar{4}$ without inversion center. The space group $I\bar{4}$ was also found for the (Na,La)WO_4_ crystal, for which the structural parameters, including the occupancy factors for Na and La sites, were refined [62]. An incommensurate (3 + 2)D modulation was revealed for the Czochralski-grown Na_2/7_Gd_4/7_MoO_4_ samples crystallized in the superspace group *I*4¯(*α−β*0,*βα*0)00 [63].

Sodium-containing double molybdates and tungstates are obtained primarily by the Czochralski method [59,60,61,62], as well as by the hydrothermal [58] and vertical Bridgman [64] methods.

### 2.2. Eulytin Family Compounds

Bismuth orthogermanate Bi_4_Ge_3_O_12_ (BGO) crystallizes in the eulytin Bi_4_(SiO_4_)_3_ structure. BGO is a self-activated-type scintillator applied in particle scintillation detectors for nuclear, space, and high-energy physics and high-resolution positron emission tomography [65,66]. It is also used as a functional material to create straight waveguides operating in the visible, mid-infrared, and telecommunication band [67,68]. Reddish BGO crystal, grown by vertical Bridgman method, with a significant emission band centered at about 1495 nm is useful in amplifier for signals in the low loss S band (1420–1520 nm) and O band (1280–1320 nm) of silica fiber and also may be used as a cryogenic scintillator for experimental rare-event research [69,70,71].

Activation of a BGO crystal by rare-earth ions, in particular, Nd^3+^, Er^3+^, Pr^3+^, Yb^3+^, Eu^3+^, Gd^3+^ ions, makes it a promising laser medium operating in different spectral regions. Doping with the Dy^3+^ ions can improve the light yield of luminescent BGO material and provide a yellowish-white light applied in solid-state lasers in visible (574 nm), near IR (1300 nm), and mid-IR (2.8–3.2 µm, 4–4.7 µm, 5.4–6 µm) spectral ranges [72,73]. Yukhin et al. [74], based only on the geometric factor, i.e., close ionic radii of Bi^3+^ and Nd^3+^ ions, concluded that Nd^3+^ ions isomorphically replace for the Bi^3+^ ions in the BGO structure. EPR study of BGO:*RE^3+^* crystals showed that all *RE^3+^* ions occupy the Bi^3+^ site without the need for charge compensation [75,76]. Based on the energies calculated from the Raman spectra for the BGO crystal, Yu et al. [70] concluded that the most favorable intrinsic defect would be the Bi/Ge antisite. Moreover, Yu et al. [70] supposed that the color of the red BGO is probably due to the OH^−^ from H_2_O appeared during the vertical Bridgman growth in the air atmosphere and/or color centers; OH^−^ may substitute an O^2−^ sitting near an antisite tetrahedrally coordinated Bi_Ge_ in the red BGO.

Single-crystal BGO, both nominally pure and doped with *RE*^3+^ ions, are generally grown from melts by the classical and low-gradient Czochralski method [77], as well as by both vertical and horizontal Bridgman method [70,78]. During the growth of BGO crystals in the air atmosphere, the water molecules present in the atmosphere dissociate into OH^−^ and H^+^ at the high temperature, and then the OH^−^ can easily enter the crystals [79]. Yu et al. [69] obtained BGO crystals of different colors depending on the composition of raw material powders by the vertical Bridgman method: ordinary transparent BGO–the sintered BGO raw material powders were put into Pt crucible immediately; red BGO (reddish color)–2 mol.% (relative to GeO_2_) H_2_O was added to the BGO raw material mixture; white (semitransparent) BGO–0.5 mol.% (relative to GeO_2_) H_2_O was added to the raw materials. The reddish BGO samples were also grown by Dunaeva et al. [73] by additional high-temperature treatment (900–950 °C) of colorless samples grown by the Czochralski method (growth atmosphere is not given).

### 2.3. Perovskite Family Compounds and Others

Cesium cadmium halides CsCd*X*_3_ (*X* = Cl, Br) with a perovskite-like structure and thulium cadmium halides TlCd*X*_3_ (*X* = Cl, I) are primarily used as laser or luminescent materials [80,81]. They are promising materials for a large number of optical applications, in particular, in communication systems as broadband amplifiers and lasers [81,82].

#### 2.3.1. CsCdX_3_ and CsCd*X_3_*:Bi (*X* = Cl, Br)

CsCd*X*_3_ crystals (*X* = Cl, Br) are promising materials for the creation of new fiber broadband tunable radiation sources, solid-state lasers, and optical amplifiers in a near-infrared (IR) spectral range (1000–1700 nm) [81]. In this range, there are narrow lines of amplification and laser generation of optical fibers with a core made from crystals doped with impurity ions.

In the CsCdBr_3_:Pr^3+^ [83] (r_Pr_^VI^ = 0.99 Å; hereinafter [84]), CsCdBr_3_:Ho^3+^ [85] (r_Ho_^VI^ = 0.90 Å), CsCdBr_3_:Tm^3+^ (r_Tm_^VI^ = 0.88 Å) [86], CsCdBr_3_: Yb^3+^ (r_Yb_^VI^ = 0.87 Å) [87] crystal structures, a replacement of Cd^2+^ cations by *RE*^3+^ ones in quasi-one-dimensional linear dimer chains (*RE*^3+^–V_Cd_″–*RE*^3+^) (V—vacancies) was found by EPR and selective excitation and fluorescence spectroscopy. The formation of asymmetric dimers, in which two *RE*^3+^ ions replace the neighboring Cd^2+^ ions and the Cd^2+^ vacancy (V_Cd_″) is located near one of the rare-earth ions, is also possible [85]. A comparison of energies of lower excited levels of isolated ion and symmetric dimer with the data obtained for a similar sample by laser selective excitation spectroscopy made it possible to uniquely identify optical spectra and conclude that the center responsible for up-conversion luminescence in CsCdBr_3_:Tm^3+^ crystals is the symmetric dimer Tm^3+^–V_Cd_″–Tm^3+^ [86].

Progress in the study of the 1000–1700 nm spectral range can be achieved by using Bi-doped crystals as an optical medium since an intense long-lived broadband luminescence in the near-IR region is observed in these materials [88]. Spectral properties of halide crystals doped with Bi ions are studied in a few works [88,89,90]. However, the obtained functional parameters are interpreted based on limited isomorphic concepts: a replacement of Cs^+^ ions by the Bi^+^ ones in the CsCdBr_3_ and CsCdCl_3_ crystalline matrices is assumed based only on the close ionic radii and charges for Bi^+^ and Cs^+^. This statement is not confirmed by any structural studies and may lead to false conclusions. Thus, a location of Bi^+^ cation, as well as its formal charge, in the CsCd*X*_3_ (*X* = Cl, Br) crystal structures has not yet been finally established.

Single-crystal CsCdBr_3_ and CsCdCl_3_, both nominally pure and doped with impurity ions, are obtained by the Bridgman method [81,90].

#### 2.3.2. TlCdX_3_ and TlCd*X_3_*:Bi (*X* = Cl, I)

TlCd*X*_3_ crystals (*X* = Cl, I) attract attention due to their luminescent and promising scintillation properties and are used for various X-ray and gamma-ray detection applications [80]. The presence of Tl^+^ ion in the host lattice makes such compounds very attractive due to their intrinsic luminescence, high effective atomic number, and density. Bi-doped TlCd*X*_3_ (*X* = Cl, I) produced broadband photoluminescence in the near-IR range have the possibility of control over optical signals at wavelengths corresponding to the telecommunication window and may be promising materials for application in optical data storage devices [82,91].

Optical and luminescent properties observed in Bi-doped TlCd*X_3_* crystals (*X* = Cl, I) were studied by Romanov et al. [82] and Vtyurina et al. [91]. However, the cause of the appearance of spectral bands in TlCdX_3_:Bi (X = Cl, I), as well as in CsCd*X_3_*:Bi (*X* = Cl, Br), are not established. Two Bi-containing luminescence impurity centers were observed in TlCdCl_3_:Bi; one of these was found to be the bismuth Bi^+^ monocation, emitting at 1025 nm [91]. In contrast, in the TlCdI_3_;Bi crystal, the luminescence center observed at a wavelength of 1175 nm is presumably related to Bi^2+^ ions rather than monovalent bismuth cations [82]. The location of Bi cations in the TlCdCl_3_ and TlCdI_3_ crystal structures is only assumed based only on the close ionic radii of Tl^+^ and Bi^+^ [82,90,91]. Thus, any confirmation of the proposed isomorphic substitution or consideration of other possible locations of the Bi cation, as well as an establishment of its real formal charge, in TlCd*X_3_* crystal structures (*X* = Cl, I) is absent in the literature.

Single-crystal TlCdCl_3_ and TlCdI_3_, both nominally pure and doped with impurity ions, are obtained by the Bridgman method [82,90,91].

## 3. Growth and Investigation Methods

### 3.1. Crystal Growth

The crystals addressed in this review, namely, the scheelite family compounds with the initial compositions PbMoO_4_, PbWO_4_, Pb(Mo,W)O_4_ and PbMoO_4_:Nd^3+^ [92,93,94], SrMoO_4_, SrMoO_4_:Ho*^3+^*and SrMoO_4_:Tm^3*+*^ [52,95,96], (Na_0.5_Gd_0.5_)WO_4_, (Na_0.5_Gd_0.5_)MoO_4_, (Na_2/7_Gd_4/7_⎕_1/7_)MoO_4_ (⎕-vacancies), (Na_6/15_Gd_8/15_⎕_1/15_)MoO_4_, (Na_0.5_La_0.5_)MoO_4_, (Na_0.5_La_0.5_)WO_4_ and (Na_0.5_Gd_0.5_)MoO_4_:Yb [97,98,99,100] and eulytin family compounds Bi_4_Ge_3_O_12_ and Bi_4_Ge_3_O_12_:Dy [73] were grown by the Czochralski method. Perovskite family compounds CsCdBr_3_ and CsCd*X*_3_:Bi (*X* = Cl, Br) [101,102] and TlCd*X_3_*:Bi (*X =* Cl, I) [82,91] were obtained by the Bridgman–Stockbarger technique.

#### 3.1.1. Scheelite Family Compounds

Nominally pure Pb*T*O_4_ (*T* = Mo, W) and Nd-doped PbMoO_4_ crystals with a diameter up to 20 mm and a length up to 60 mm were grown by the Czochralski method (ceramic technology) on the “Analog” growth equipment, provided with a weight sensor, in the Pt crucible. The synthesis was carried out using a ceramic technology from the corresponding oxides taken in a stoichiometric ratio or with an excess of PbO [92,93,103,104]. The Nd^3+^ ions were introduced into the PbMoO_4_ structure as pre-synthesized compounds Nd_2_O_3_, Nd_2_(MoO_4_)_3_, NaNd(MoO_4_)_2_ and NdNbO_4_ over PbMoO_4_ stoichiometry. PbMoO_4_, PbMoO_4_:Nd_2_O_3_ and PbMoO_4_:NdNbO_4_ crystals were grown from melt with stoichiometric PbO/MoO_3_ ratio, whereas PbMoO_4_:Nd_2_(MoO_4_)_3_ and PbMoO_4_:NaNd(MoO_4_)_2_ crystals were obtained from melt enriched with MoO_3_ [103]. The growth parameters for all the single crystals were as follows: crystal growth was carried out on a seed oriented along the direction <001>; crystallization front was flat or slightly convex; temperature gradient *T*_z_ = 50–70 °C cm^−1^; rotation rate ω = 20–30 rpm; pulling rate *V*_z_ = 1–3 mm h^−1^. All the crystals were free of impurity phases and macroscale defects, in particular, gas bubbles and crystal cracking.

Single-crystal solid solutions PbMo*_x_*W_1−*x*_O_4_ with a diameter of 15–20 mm and a length of 60 mm were grown by the Czochralski method in air under conditions similar to those described above [105]. The over-stoichiometric amount of YNbO_4_ (15 wt%) was added to the initial charge. Extra pure grade PbO, WO_3_, Nb_2_O_5_ and Y_2_O_3_ and GR grade MoO_3_ oxides were taken for initial charge preparation.

Optically homogeneous SrMoO_4_, both nominally pure and doped with Tm^3+^ and Ho^3+^ ions, were grown by the Czochralski method (Pt crucible; air atmosphere; SrMoO_4_ seed; seed orientation <100>) [95]. Extra pure grade (5 N) SrCO_3_, MoO_3_, Tm_2_O_3_, Ho_2_O_3_, and Nb_2_O_5_ powders were used as starting materials. The SrMoO_4_ charge was obtained from SrCO_3_ and MoO_3_ by solid-phase synthesis at 1140 °C for 5 h. Dopant ions were added into the melt in the form of *RE*Nb^5+^O_4_ (*RE*^3*+*^ = Tm, Ho) over SrMoO_4_ stoichiometry. On one hand, this ensures the electroneutrality of the system (Sr^2+^ → *RE*^3+^, Mo^6+^ → Nb^5+^), and, on the other hand, is favorable according to the isomorphism theory: *RE*Nb^5+^O_4_ crystallizes in the monoclinic fergusonite-type structure with the space group *C*2/*c* [106]. To obtain crystals of high optical quality, the bulk crystallization rate was decreased from 0.5 to 0.05 cm^3^ h^−1^ with increasing dopant concentration in the melt from 0.1 wt% to 3 wt%. The axial thermal gradient was 100 °C cm^−1^ in the growth zone and 15 °C cm^−1^ in the annealing zone. The cooling rate of the crystal in the annealing zone was 5 °C min^−1^. The as-grown single-crystal boules with up to 20 mm and a length up to 80 mm were optically transparent. Nominally pure and Tm-doped SrMoO_4_ crystals were colorless and Ho-doped ones were light-yellow.

Single crystals with the nominal (charge) compositions (Na_0.5_Gd_0.5_)WO_4_, (Na_0.5_Gd_0.5_)MoO_4_, (Na_2/7_Gd_4/7_⎕_1/7_)MoO_4_ (⎕-vacancies), (Na_6/15_Gd_8/15_⎕_1/15_)MoO_4_, (Na_0.5_La_0.5_)MoO_4_, (Na_0.5_La_0.5_)WO_4_ and Na_0.5_Gd_0.5_)MoO_4_:Yb were grown by the Czochralski method from a Pt crucible in air or Ar (sample *NGM*-Ar) atmosphere [57,98,99,107,108,109]. The pulling rate at different stages of crystal growth process was varied to provide the actual velocity of crystallization front moving at the level of 1.2 mm h^−1^ regardless of the stage of the process (growth cone or cylindrical part), taking into account the gradual lowering of the melt level in the crucible. The rotation rate was 8 rpm. The charge for crystal growth was prepared from the required amounts of pre-dried initial chemicals Na_2_CO_3_ (99.5%), Gd_2_O_3_ (99,999%) and MoO_3_ (99.99%), which were thoroughly mixed and sintered in a muffle furnace at 700 °C for 12 h. To reduce crystal cracking during the growth process, the as-grown boule moved into the resistive furnace mounted directly above the crucible and heated up to 700 °C. The viewing window, made in ceramic heat-shields, was covered by a transparent quartz glass plate. After the crystal growth process, several crystals grown in the air were additionally annealed in air at 800 °C for 4 days.

#### 3.1.2. Eulytin Family Compounds

Colorless optically homogeneous Bi_4_Ge_3_O_12_ single crystals both nominally pure (BGO) and doped with Dy_2_O_3_ (BGO:Dy) of 16 mm in diameter and 65 mm in length were grown by the Czochralski method [110]. Special growth and weight control PC program AURA at NIKA-3 growth setup was used to obtain optical quality crystals. High-purity (4 N) Bi_2_O_3_, GeO_2_, Dy_2_O_3_ powders were used as starting materials. The growth orientation was [100]. Dopants were added into the melt in the oxide form in the concentrations of 0.1 wt% and 1.0 wt% Dy_2_O_3_. The axial thermal gradient was 80–90 °C cm^−1^ in the growth zone and 10–15 °C cm^−1^ in the annealing zone. The optimal bulk crystallization rate was estimated to be 0.1 cm^3^ h^−1^. The as-grown crystals were transparent in the spectral range of 0.35–6.5 mm, free of cracks, bubbles, inclusions of second phases, and scattering centers. The isomorphic capacity of Dy^3+^ ions in the matrix does not exceed 0.35 at%. Further increasing of Dy^3+^ dopant content leads to phase heterogeneity of as-grown crystal, in particular, inclusions of the “second” phase in the sample body.

The colored (pink) samples BGO(P) were obtained by additional high-temperature treatment (900–950 °C) of plates of 1.5–2.0 mm in thickness, cut from the colorless boule, in graphite crucibles at a pressure of 10^−2^ Torr and using several milligrams of stannous oxalate SnC_2_O_4_ as a reducing agent. The annealing time was varied from 6 to 10 h [110].

#### 3.1.3. Perovskite Family Compounds and Others

For the Bi-doped CsCdCl_3_ (CsCdCl_3_:Bi) and CsCdBr_3_ (CsCdBr_3_:Bi) growth, double chlorides and bromides taken in a molar ratio of CdCl_2_:CsCl:BiCl_3_ = 59.8:39.8:0.4 and CdBr_2_:CsBr:BiBr_3_ = 54.5:44.5:1, respectively, were used [111]. A metallic bismuth was added in a molar ratio of Bi_Me_/BiCl_3_ = 0.03 and Bi_Me_/BiBr_3_ = 1, assuming a formation of Bi^1+^ ions. Due to the hygroscopic nature of reagents, all the procedures such as weighing and mixing of components and their placing into a quartz ampoule of 24 mm in diameter were carried out in an argon-filled Labconco glove box. The filled ampoule was removed from the box, pumped out to the fore vacuum, blown with helium, re-pumped out, and then sealed off at the preformed waist.

The nominally pure CsCdBr_3_ and Bi-doped CsCdBr_3_ (a yellow-green color) and CsCdCl_3_ (pale green-blue color) single crystals were grown by the Bridgman–Stockbarger technique at an ampule lowering rate of 2 mm h^−1^ [111]. The temperatures in the upper and lower zones of the furnace were, respectively, 580 and 530 °C for the CsCdCl_3_:Bi and 450 and 380 °C for the CsCdBr_3_:Bi. After completion of the growth process, the crystals were cooled to room temperature at a rate of 0.33 °C min^−1^.

The initial batch for growth of TlCdCl_3_:Bi crystal was composed of the TlCl, CdCl_2_, and BiCl_3_ chlorides taken in a molar ratio of 44.8:54.8:0.4, respectively. The initial batch contains a small amount of metallic bismuth taken in proportion BiCl_3_/Bi = 1. During crystal growth by the Bridgman–Stockbarger method, the temperature in the upper zone of the furnace was maintained at 733 K (460 °C) [112]. According to the energy-dispersive X-ray microanalysis, the Tl, Cd and Cl content are 20.11 at%, 17.63 at%, 58.74 at%, respectively. According to inductively coupled plasma mass spectrometry data, the content of bismuth impurity in the resulting sample is 0.1 at%.

The initial batch for growth of TlCdI_3_:Bi crystal by the Bridgman–Stockbarger method was composed of the TlI, CdI_2_, and BiI_3_ iodides taken in a molar ratio of 49.9:49.9:0.2, respectively. Initial components and a small amount of metallic bismuth taken in proportion BiI_3_/Bi = 1 were placed into a quartz ampoule of 20 mm in diameter [112]. All the procedures such as weighing and mixing of components and their placing into a quartz ampoule were carried out in an atmosphere of dry inert gas. The filled ampoule was pumped out to the fore vacuum and then sealed off at the preformed waist. The single-crystal grew at an ampoule lowering speed of 1 mm h^−1^. The as-grown TlCdI_3_:Bi single crystal with a diameter of 20 mm and a length of 100 mm was dark red.

### 3.2. Characterization Methods

The crystals addressed in this review were studied using single-crystal neutron and X-ray diffraction and X-ray Absorption Spectroscopy.

#### 3.2.1. Single-Crystal X-Ray and Neutron Diffraction

X-ray diffraction analysis (XRD) of PbMoO_4_, PbWO_4_, PbMoO_4_:Nd^3+^, PbMo_x_W_1−x_O_4_, (Na_0.5_Gd_0.5_)MoO_4_:Yb and Bi_4_Ge_3_O_12_:Dy microcrystals ~0.2 × 0.2 × 0.2 mm^3^ in size was carried out on a STOE STADIVARI PILATUS-100 K single-crystal diffractometer (STOE & Cie GmbH, Darmstadt, Germany) at room temperature (Mo*K*_α_ or/and Ag*K*_α_; graphite monochromator; ω/φ-scan mode). The SrMoO_4_, SrMoO_4_:Ho^3+^, SrMoO_4_:Tm^3+^, (Na_0.5_Gd_0.5_)WO_4_, (Na_0.5_Gd_0.5_)MoO_4_, (Na_2/7_Gd_4/7_⎕_1/7_)MoO_4_ (⎕-vacancies), (Na_6/15_Gd_8/15_⎕_1/15_)MoO_4_, (Na_0.5_La_0.5_)MoO_4_, (Na_0.5_La_0.5_)WO_4_, Bi_4_Ge_3_O_12_, CsCdBr_3_, CsCdBr_3_:Bi, CsCdCl_3_:Bi, TlCdCl_3_:Bi and TlCdI_3_:Bi microcrystals ~0.2 × 0.2 × 0.2 mm^3^ in size were studied using an Enraf-Nonius CAD4 single-crystal diffractometer (Enraf-Nonius, Rotterdam, Netherlands) at room temperature (Mo*K*_α_ or Ag*K*_α_; graphite monochromator; ω-scan mode). To reduce an error associated with the absorption, the XRD data were collected over the entire Ewald sphere.

Neutron diffraction analysis (ND) of PbMoO_4_, PbWO_4_, Pb(Mo,W)O_4_ and PbMoO_4_:Nd^3+^ crystal macroparts ~5 × 5 × 5 mm^3^ in size was carried out at room temperature on the four-circle single-crystal diffractometer installed at the hot source (5C2) of the Orphee reactor (Laboratoire Léon Brillouin, Gif sur Yvette Cedex, France; λ = 0.83 Å; ω-scan mode).

The preliminary diffraction data processing was carried out using the WinGX pack [113] with a correction for absorption (MULTISCAN or ψ-scan). The atomic coordinates, anisotropic displacement parameters of all atoms and occupancies of cation and oxygen sites were refined using the SHELXL-97, SHELXL-14 or SHELXL-2015 software packages [114], taking into account the atomic scattering curves for neutral atoms, with semi-empirical (azimuthal scan) [115] or empirical [116] correction for absorption.

The structural parameters were refined in several steps. Initially, the positional and thermal parameters were simultaneously refined in isotropic and anisotropic approximations with fixed occupancies of all sites. Then, simultaneously with the thermal parameters, the occupancy factor of first cation site was refined with fixed occupancies of other sites, then the occupancy of second cation site was refined with fixed occupancies of other sites, and finally, the occupancy of anion site was refined with fixed occupancies of cation sites. The strategy and tactics of refining the occupancies of crystallographic sites were different depending on the structure and composition of the compounds. Due to the well-known correlation between thermal parameters and site occupancies we used the strategy of crystal structure refinement developed by us for both present objects and other complex oxides and described in detail in Refs [7,117]. After each refinement step, the residual electron density, atomic displacements, and interatomic distances were analyzed. The actual compositions taking into account the electroneutrality, the correct values of the atomic displacement parameters, the lowest values of the *R* factors, and the absence of residual electron-density peaks serve as criteria for the accuracy of the structure refinement and the correctness of the determination of the composition.

#### 3.2.2. X-Ray Absorption Spectroscopy

X-ray absorption spectroscopy (EXAFS/XANES) measurements of powdered SrMoO_4_, SrMoO_4_:Ho^3+^, SrMoO_4_:Tm^3+^, Bi_4_Ge_3_O_12_:Dy, CsCdBr_3_, CsCdBr_3_:Bi, CsCdCl_3_:Bi, TlCdCl_3_:Bi and TlCdI_3_:Bi single crystals (~100 mg), taken from the same parts of the crystalline boule as the microparts for the diffraction experiment, were performed at room temperature at the Structural Materials Science end-station installed at the Kurchatov Synchrotron Radiation Source (NRC “Kurchatov Institute”, Moscow, Russia) [118].

The energy scans were performed using Si(220)-crystal monochromator with the energy resolution ΔE/E ~2·10^−4^. EXAFS spectra were collected in a transmission mode, placing the sample between two ionization chambers connected to picoammeter (Keithley, Solon, OH, USA), which also serves as a voltage source. The intensity of the monochromatic beam, incident on the sample and passing through it, was measured in the air ionization chamber and chamber, filled with pure Ar up to atmospheric pressure, respectively. Samples were evenly applied on the adhesive tape, having a small X-ray absorption coefficient.

The standard processing of experimental spectra was performed using IFEFFIT program package [119]. A character of the atom’s nearest environment was revealed by analyzing the radial distribution function φ(*r*), obtained by Fourier transform of *k*^3^·χ(*k*) function, where the multiplication by *k*^3^ was used to compensate attenuation of Fourier transforms with distance from the absorption edge. Fourier transforms of EXAFS oscillations were extracted in the range of photoelectron wave number (*k*) from 2 to 12.5 Å^−1^ (SrMoO_4_, SrMoO_4_:Ho^3+^ and SrMoO_4_:Tm^3+^), from 2 to 6 Å^−1^ (Bi_4_Ge_3_O_12_:Dy), from 2 to 12.0 Å^−1^ (CsCdBr_3_ and CsCdBr_3_:Bi, TlCdCl_3_:Bi and TlCdI_3_:Bi) and modeled in the range of interatomic distance (*d*_EXAFS_, Å).

### 3.3. Functional Properties

The absorption spectra crystals were measured for SrMoO_4_:Tm^3+^ at room temperature [95].

The transmission, spectral-luminescence, and excitation spectra were studied for nominally pure Bi_4_Ge_3_O_12_ [110], whereas spectral-luminescence and absorption spectra were investigated for BGO:1.0% Dy [120].

Photoluminescence (PL) spectra and photoluminescence decay kinetics were studied for the CsCdBr_3_:Bi [111], CsCdCl_3_:Bi [101], TlCdCl_3_:Bi and TlCdI_3_:Bi [112] samples.

Impedance spectroscopy investigations, namely the temperature and frequency dependences of the capacitance *C(f, T)* and the conductance *G(f,T)* in the frequency range from 25 to 106 Hz and the temperature range from 100 to 500 °C (E7 20 *RLC* meter), were performed for PbMoO_4_:Nd^3+^ [103].

## 4. Structural Features in Functional Crystals of Complex Compositions

### 4.1. Scheelite Family Compounds: PbTO_4_, SrMoO_4_, (Na,RE^3+^)TO_4_ (T = Mo, W; RE^3+^ = La,Gd)

Compounds with scheelite or scheelite-like structures constitute an extensive class of objects with simple (e.g., PbTO_4_, SrMoO_4_) or complex (e.g., (Na,RE)TO_4_) compositions, having centrosymmetric scheelite (space group *I*4_1_/*a*) or noncentrosymmetric structures [121,122].

**Figure 1:**
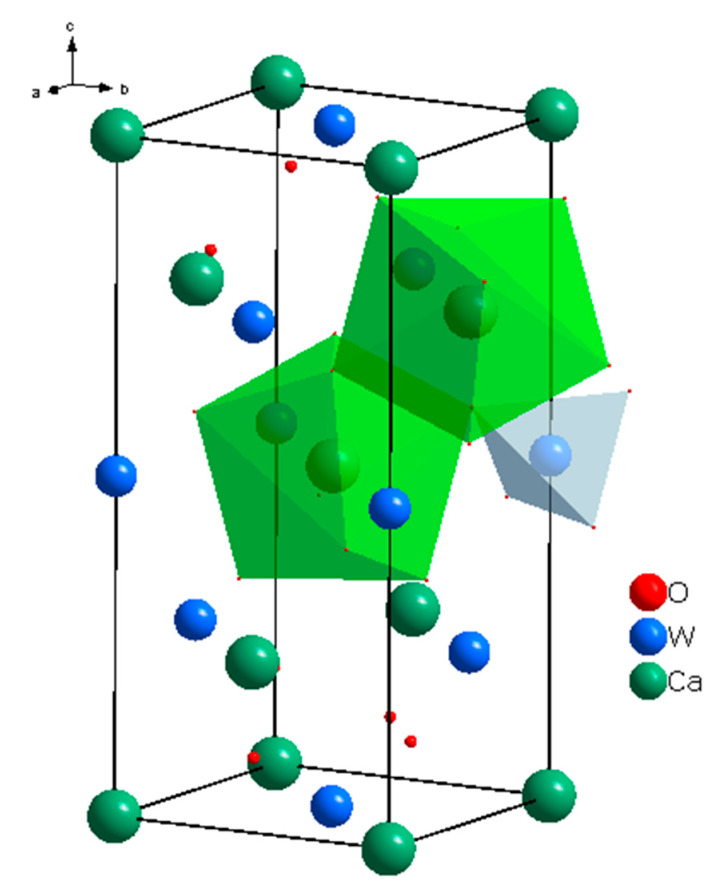
Crystal structure of scheelite CaWO_4_.

In the scheelite CaWO_4_ crystal structure (space group *I*4_1_/*a*, *Z* = 4), the Ca cations occupy the Wyckoff site 4*a* with coordinates 0 0 0.5 forming the dodecahedra with refracted upper and lower faces with two different Ca–O interatomic distances with CN Ca = 4 + 4 (CN is a coordination number) (Figure 1). The W^6*+*^ cations occupy the Wyckoff site 4*b* with coordinates 0 0 0 and form elongated tetrahedra with four equal W–O distances (CN W = 4). The O^2−^ ions are coordinated, in turn, by two Ca atoms and one W atom and occupy general site 16*c* with coordinates *x y z*. The CaWO_4_ structure can be classified as a quasi-layer structure: the O atoms from close WO_4_ tetrahedra form layers.

The CaO_8_ polyhedra are connected by the edges, each polyhedron being surrounded by four neighboring CaO_8_-polyhedra. Each cation is surrounded by four similar cations along the tetrahedron and eight cations of another element along the remaining vertices of the cuboctahedron. As a result of such a mutual arrangement of compact oxygen networks, differentiation of the cation environment occurs. In planes, parallel to the square faces of the cationic cuboctahedron, a checkerboard ordering of Ca and W atoms is observed.

#### 4.1.1. Pb*T*O_4_ (*T* = Mo,W), Pb(Mo,W)O_4_, PbMoO_4_:Nd^3+^

##### Pb*T*O_4_ (*T* = Mo,W)

PbMoO_4_ (PMO) and PbWO_4_ (PWO) crystals were grown by the Czochralski method from a stoichiometric mixture of the corresponding oxides (PbO + MoO_3_/WO_3_) using different growth atmosphere (air–A, PTO-A; nitrogen–N_2_, PTO-N_2_) with subsequent post-growth annealing in vacuum [104]. According to XRD (Table 1), the growth atmosphere affects the composition of the obtained PMO-A and PMO-N_2_ crystals: the deficiency of the dodecahedral site is greater for crystals grown in a nitrogen atmosphere–PMO-N_2_.

Self-compensation of electric charges for these crystals can be described by a quasi-chemical reaction, taking into account their color (Table 1): 0 → V_Pb_^n^′ + nh^•^ → (V_Pb_^n^′,nh^•^)^x^ (Equation (1)), where (V_Pb_^n^′,nh^•^)^x^ is a color center or 0 → V_Pb_^n^′ + nPb_Pb_^•^ (Equation (2)), i.e., “holes” nh^•^ are localized on Pb^2+^ ions with a partial transition Pb^2+^ → Pb^3+^. A distinctive feature of PWO-A and PWO-N_2_ crystals is the presence of a great amount of oxygen vacancies: 0 → V_O_^n•^ + ne′ → (V_O_^n•^,ne′)^×^ with color center (V_O_^n•^,ne′)^×^ (Equation (3)). According to XRD data for PWO-N_2_ crystals, vacancies in the W site are possible: 0 → V_W_^m^′ + V_O_^p•^ + ne′ → V_W_^m^′ + V_O_^m•^ + (V_O_^n•^,ne′)^×^ (Equation (4)). The transition W^6+^ → W^5+^ is unlikely since it should be accompanied by an appearance of a color associated with W^5+^ ions. PMO crystal, grown in a nitrogen atmosphere with an excess of lead [104], have the refined composition (ND) PbMo^5.90(4)+^(O_3.95(2)_⎕_0.05_) (⎕-vacancies). It can be described by the quasi-chemical reaction 0 → V_O_^p•^ + pMo_Mo_′ (Equation (5)), i.e., “electrons” ne′ are localized on Mo^6+^ ions with a partial transition Mo^6+^ → Mo^5+^ and appearance of a gray-violet coloration due to Mo^5+^ ions. Hereinafter, point defects and quasi-chemical reactions are designated according to Kröger–Vink notation [123] and guidance given in Ref. [124], respectively.

The XRD analysis of microparts of PWO-A and PWO-N_2_ crystals revealed the presence of additional reflections (~50%), which cannot be indexed within the framework of space group *I*4_1_*/a* (Table 1). It indicates a decrease in the symmetry of crystals to space group $I\bar{4}$. A selection of the space group $I\bar{4}$ from three possible space groups *I*4*/m, I*4 or $I\bar{4}$ based on the extinction law is due to the correspondence of regular system of points of space groups *I*4_1_*/a* (scheelite structure) and $I\bar{4}$. This effect, which was not revealed for PMO crystals (Table 1), was observed for PWO for the first time.

The refinement of the composition of PWO-N_2_ crystal (XRD) within the framework of space group $I\bar{4}$ allows writing its composition as [Pb(1)_0.500(2)_W(1)_0.500(4)_][(Pb(2)_0.500(2)_W(2)_0.492(4)_] [O(1)_4.000_O(2)_3.976(24)_]. The change in symmetry is accompanied by the splitting of 4-fold Pb and W sites and 8-fold O site (space group *I*4_1_*/a*) into two 2-fold Pb and W sites and two 4-fold O sites (space group $I\bar{4}$), respectively.

Different symmetry of scheelite family molybdates and tungstates was found for complex oxides with the general composition CaGd_2(1−*x*)_Eu_2*x*_(MoO_4_)_4(1−*y*)_(WO_4_)_4*y*_ (0 ≤ *x* ≤ 1, 0 ≤ *y* ≤ 1) by electron transmission microscopy and powder X-ray diffraction [125]. A solid solution with *y* = 0 (molybdates) has (3 + 2)D incommensurately modulated structure and crystallizes in the framework of superspace group *I*4_1_/*a*(*α,β*,0)00(*−β,α*,0)00. The structure with *y* = 1 (tungstates) is (3 + 1)D incommensurately modulated with superspace group *I*2/*b*(*αβ*0)00 [125].

##### Pb(Mo,W)O_4_ Solid Solutions

Single-crystal PbMo*_x_*W_1-*x*_O_4_ (PMWO) solid solutions (*x* = 0.2, 0.5, 0.8) were obtained by the Czochralski method in air atmosphere from initial charge with over-stoichiometric amount of YNbO_4_ (15 wt%) [105]. The additional introduction of YNbO_4_ to the initial mixture (PbO + MoO_3_/WO_3_) prevents the formation of defects (Pb^2+^,Y^3+^)(Mo^6+^,W^6+^,Nb^5+^)O_4_ and ensures the electroneutrality of the system. It is also favorable for the isomorphic miscibility of PbMO_4_ and YNbO_4_, since these phases belong to the same scheelite family [4,126].

According to the ND and XRD data [105], in the structures of all PbMo*_x_*W_1−*x*_O_4_ solid solutions, Pb^2+^ ions are partially replaced by Y^3+^ ions (r_Y_^3+ VIII^ = 1.02 Å, r_Pb_^2+ VIII^ = 1.29 Å [84]; Δr/r_min_ = (r_Pb_-r_Y_)/r_Y_ ~26%). Moreover, an increase in *x* increases the content of Y^3+^ ions (despite the fact that their content in the charge is the same for all solid solutions) and oxygen vacancies (XRD) with the highest content in PMWO-3 crystal (XRD, ND) (Table 2).

It should be noted that the site occupancy is proportional to the form factor of the atom, which in turn is determined by the element number (N_Pb_ = 82, N_Y_ = 39; N_W_ = 74, N_Mo_ = 42, N_Nb_ = 41). Therefore, it is not possible to determine the content of Nb^5+^ by XRD. However, the nuclear scattering factors of Mo (*b* = 0.695) and Nb (*b* = 0.705) are slightly different, which allowed to estimate the content of Mo, W and Nb in the tetrahedral site of structures of all solid solutions by the ND analysis (Table 2). In this case, the content of Nb^5+^ ions correlates with the content of Y^3+^ ions. According to the results of structural analysis, the defect formation in solid solutions can be written in the general form as 0 → Y_Pb_^•^ + Nb_(W,Mo)_′ + V_O_^n•^ (Equation (6)), which confirms the colorlessness of these crystals by the absence of a color center.

It is interesting to examine the change in unit cell parameters and unit cell volumes as indicators of compositions of Pb^2+^Mo^6+^*_x_*W^6+^_1−*x*_O_4_ solid solutions. According to ND data, with increasing *x*, the *a* unit cell parameter and the cell volume decreases, while the change in *c* unit cell parameter can be described by bell profile with a maximum at *x* = 0.5. At the same time, according to XRD data, with increasing *x*, both *a* and *c* parameters change along bell profile, but with a minimum and a maximum at *x* = 0.5 for the *a* and *c* parameter, respectively [105]. Such a different character of dependences indicates the heterogeneity in the composition of single-crystal PMWO boules, on one hand, and d the opposing actions of ions with different radii (r_Nb_^5 + IV^ = 0.48 Å, r_Mo_^6 + IV^ = 0.41 Å, r_W_^6 + IV^ = 0.44 Å; r_Nb_^5+^ > r_W_^6+^ > r_Mo_^6+^), on the other hand.

An introduction of over-stoichiometric buffer component Y^3+^Nb^5+^O_4_ into the charge Pb^2+^Mo^6+^*_x_*W^6+^_1−*x*_O_4_ with *x* = 0.2, 0.5, and 0.8 contributes to the growth of colorless crystals (i.e., without color centers). In this case, the assumption made by Oeder et al. [127] that the yellow color of crystals grown by the Czochralski method is related to contamination with crucible material (Pt) is not confirmed. Despite the presence of oxygen vacancies in the structures of almost all solid solutions (Table 2), no symmetry change was observed, which is most likely due to a disorder of tetrahedral site with the arrangement of three Mo, W, and Nb atoms in it.

##### PbMoO_4_:Nd^3+^

Nominally pure PbMoO_4_ and Nd^3+^-doped PbMoO_4_ crystals were grown by the Czochralski method in inert and oxidizing atmospheres, respectively. The over-stoichiometric Nd^3+^ ions were introduced into the initial charge in the form of pre-synthesized compounds: Nd_2_O_3_ (does not belong to the scheelite family), Nd_2_(MoO_4_)_3_ (defective scheelite-like structure), (Na_0.5_Nd_0.5_)MoO_4_ (scheelite structure) and NdNbO_4_ (scheelite-like structure). The PbMoO_4_, PbMoO_4_:Nd_2_O_3_ and PbMoO_4_:NdNbO_4_ crystals were grown from melt with stoichiometric PbO/MoO_3_ ratio. The PbMoO_4_:Nd_2_(MoO_4_)_3_ and PbMoO_4_:NaNd(MoO_4_)_2_ crystals were obtained from melt enriched with MoO_3_ (Table 3) [92,93,103].

According to the XRD and ND data, the composition of Sample 1 is almost stoichiometric (Table 3), if standard deviations are taken into account. Refinement of compositions of crystallographic sites in the structures of Samples 2, 4 and 5 did not reveal any deficiency of Mo and O sites. The ND analysis allowed to determine the composition of the tetrahedral site of the structure of Sample 3–(Pb^2+^_0.968(16)_Nd^3+^_0.032_)(Mo^6+^_0.970(20)_Nb^5+^_0.030_)O_4_ (Table 3). It is necessary to pay attention to the lilac color of Sample 3, which is due only to the presence of Nd^3+^ ions and deep lilac color of Sample 5 with a complex composition of the dodecahedral site containing Nd^3+^ ions (Table 3).

According to the ND data, vacancies are observed in the dodecahedral site of structures of Samples 2 and 4 with the implementation of electroneutrality condition. Taking into account the color of Samples 2 and 4 (Table 3), a quasi-chemical reaction can be written as 0 → V_Pb_^m^′ + qNd_Pb_^•^ + nh^•^ → V_Pb_^p^′ + qNd_Pb_^•^ + (V_Pb_^n^′,nh^•^)^x^ (Equation (7)), where (V_Pb_^n′^,nh^•^)^x^ is a color center or 0 → V_Pb_^n^′ + nPb_Pb_^•^ (Equation (2)),, i.e., “holes” nh^•^ are localized on Pb^2+^ ions with a partial transition Pb^2+^ → Pb^3+^.

A structural study of scheelite family PbMoO_4_ crystals doped with Nd^3+^ ions introduced by different methods showed that the most preferable Nd-containing compounds used to PMO:Nd synthesis are (Na_0.5_Nd_0.5_)MoO_4_ with scheelite structure and NdNbO_4_ with distorted scheelite structure. They do not lead to additional optical absorption (i.e., without the formation of color centers) despite the presence of additional ions (Nb^5+^ and Na^1+^) in the compositions of Samples 3 and 5 (Table 3). The introduction of Nd^3+^ ions in the form of NdNbO_4_ made it possible to obtain the most structurally perfect crystal (Sample 3), which is confirmed by one time of Nd^3+^ luminescence decay and the minimum number of *RC* chains in equivalent electrical circuits (*RC* is an electric circuit consisting of a resistor and a capacitor) [33,103].

#### 4.1.2. SrMoO_4_ and SrMoO_4_:*RE* (*RE^3+^* = Ho,Tm)

Optically homogeneous SrMoO_4_, SrMoO_4_:Ho^3+^, and SrMoO_4_:Tm^3+^ crystals (Table 4) were grown by the Czochralski method in air atmosphere. Dopant ions were added into the melt in the form of *RE*NbO_4_ (*RE* = Tm, Ho) over SrMoO_4_ stoichiometry [95].

The XRD study of colorless SMO crystal (Table 4) indicates that it is defect-free, which is in good agreement with the published data [128,129,130,131,132,133,134].

The refinement of occupancies of Sr and Mo sites in the SMO:0.1Ho and SMO:0.5Ho structures showed a possible replacement of Sr^2+^ ions by Ho^3+^ ones. The Ho^3+^ content in SMO:0.5Ho is much higher, which is consistent with the composition of the initial charge. No vacancies were found in the remaining crystallographic sites in the SMO:0.1Ho and SMO:0.5Ho structures (within the appropriate margin of error) (Table 4). For the SMO:1.0Ho, vacancies were found in all crystallographic sites and the residual electron density with coordinates 0 ¼–0.0804, i.e., near Mo ions, was revealed. Hence, the composition of the SMO:1.0Ho crystal can be written in the form (Sr_0.998(2)_Ho_0.002_)[(Mo_0.998_⎕_0.002_)(Nb_0.002_)_i_]O_3.96(3)_, taking into account the electroneutrality of cationic sites (Table 4).

A similar refinement of site occupancies for SMO:0.5Tm microcrystal allowed to reveal vacancies in the Mo site and their absence in the O site (Table 4). Analysis of the residual electron density showed peaks with coordinates 0 ¼ 0.0905 and ½ ¾ 0.085 in the vicinity of Mo and Sr atoms, respectively, which may indicate a presence of interstitial Tm^3+^ and Nb^5+^ ions. In this case, the refined composition of SMO:0.5Tm sample can be written in the form [Sr_0.996(2)_⎕_0.004_)(Tm_0.004_)_i_][Mo_0.996(3)_⎕_0.004_)(Nb_0.004__(i)_]O_4_ (⎕-vacancies), taking into account the electroneutrality of system (Table 4).

In the structure of SMO:1.0Tm microcrystal, vacancies in the Sr, Mo, and O sites were found. The content of Sr and Mo vacancies is higher in the SMO:1.0Tm compared to SMO:0.5Tm. The presence of pronounced clots of electron density near the Sr and Mo sites allowed to refine the SMO:1.0Tm structure with interstitial Tm^3+^ and Nb^5+^ ions. General composition of SMO:1.0Tm microcrystal with compensated electroneutrality of the cationic sites can be written as [(Sr_0.992(3)_⎕_0.008_)(Tm_.008(i)_)][(Mo_0.992_⎕_0.008_)(Nb_0.008(i)_)]O_3.80(4)_ (⎕-vacancies) (Table 4).

It follows that the Ho^3+^ and Tm^3+^ ions occupy different sites in the SMO:Ho and SMO:Tm structures, which is probably due to different sizes of Sr and Ho ions (Δr/r_min_ = (r_Sr_-r_Ho_)/r_Ho_ ~23%) or Sr and Tm ones (Δr/r_min_ = (r_Sr_-r_Tm_)/r_Tm_ ~27%).

The obvious deficiency of O sites in SMO:1.0Ho and SMO:1.0Tm structures (greater for SMO:1.0Tm) confirms indirectly the presence of Nb^5+^ ions in interstitial sites with their own coordination environment with O^2−^ ions. Possible additional O(1) atoms with coordinates–0.1759 0.4351–0.0097 were detected in the residual electron density for the SMO:1.0Tm microcrystal. However, taking into account additional O(1) atoms, the total O content remains less than the stoichiometric one and the electroneutrality of the system is not observed, especially for SMO:1.0Tm (Table 4). An explanation can be obtained by studying the local structure of these objects by X-ray absorption spectroscopy.

**Figure 2:**
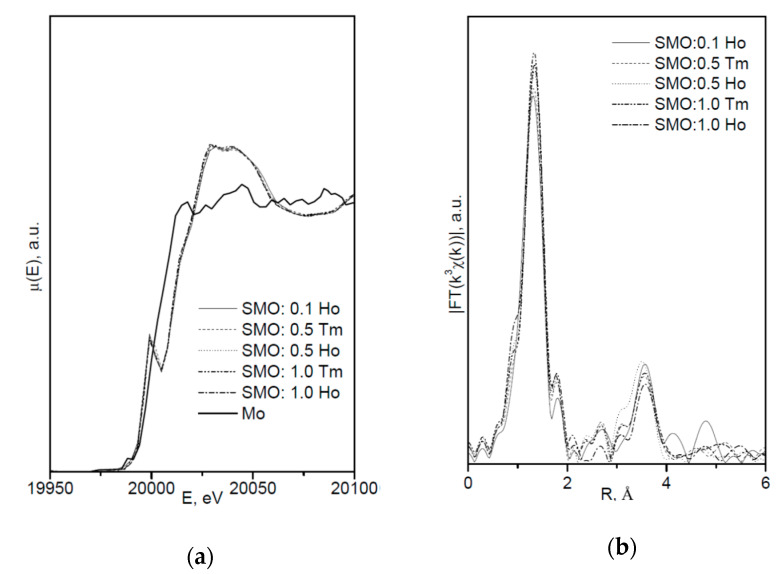
Mo K-edge-normalized (**a**) X-ray absorption spectroscopy (XANES) spectra and (**b**) EXAFS Fourier transforms for SrMoO_4_:Ho and SrMoO_4_:Tm crystals. The standard is metal molybdenum.

Figure 2 shows the XANES spectra and EXAFS Fourier transforms, measured at the Mo K-edge.

The XANES spectra of the samples differ slightly from each other (Figure 2a). Similar XANES oscillations indicate the similar coordination of both Sr^2+^ and Mo^6+^ ions in all structures. An absence of shifts in the XANES spectral curves indicates the invariance of the Sr^2+^ and Mo^6+^ formal charges in all samples.

EXAFS Fourier transforms have similar shapes and are characterized by two peaks at *R* ~1.2 and 3.6 Å (*R* is the distance from the Mo^6+^ ion to its coordination spheres) (Figure 2b). The first peak corresponds to photoelectron scattering on the nearest Mo^6+^ coordination sphere occupied by O^2−^ ions. The second peak corresponds to scattering on the distant coordination sphere occupied by Mo^6+^ and Sr^2+^ ions. The intensities of the peaks in different samples differ from each other, which indicate different coordination numbers of Mo^6+^ in the structures under investigation (Table 5).

Decreased values for CN Mo are observed for the SMO:1.0Ho and SMO:1.0Tm samples due to the presence of oxygen vacancies (Table 5). Increased Mo–O interatomic distances in doped samples compared to nominally pure SMO are due to a partial replacement of Mo^6+^ (r_Mo_^6+ IV^ = 0.41 Å [84]) ions by the Nb^5+^ ones (r_Nb_^5+ IV^ = 0.48 Å [84]).

According to joint XRD and EXAFS/XANES results, the processes of defect formation in SMO:1.0Ho and SMO:1.0Tm crystals can be written in the final form 0 → Ho_Sr_^•^ + V_Mo_^n^′ + Nb_Mo_′ + Nb_i_^m^^•^ + V_O_^p^^•^ + O_i_^k^′ (Equation (8)) and 0 → V_Sr_^q^′ + Tm_i_^s^^•^ + V_Mo_^n^′ + Nb_Mo_′ + Nb_i_^m^^•^ + V_O_^p^^•^ + O_i_^k^′ (Equation (9)), respectively (Table 4). It should be noted that the color of crystals (SMO and SMO:Tm are colorless; SMO:Ho are yellowish due to the presence of Ho^3+^ ions) confirms the proposed quasi-chemical reactions without color centers and any change in the Mo^6+^ formal charge. It follows that the color of crystals can act as an indicator of their actual compositions [103,104].

The optical absorption spectra of SMO:Tm samples contain absorption bands at wavelengths 360, 473, and 690 nm in the visible spectral region, which correspond to energy transitions ^3^*H*_6_–^1^*D*_2_, ^1^*G*_4_, ^3^*F*_2_, respectively. In the infrared region, there are absorption bands at wavelengths 795, 1214, and 1750 nm corresponding to energy transitions ^3^*H*_6_–^3^*H*_4_, ^3^*H*_5_, ^3^*F*_4_ [95,135]. The absorption increases with increasing concentration of active Tm^3+^ ions in the crystal: intrinsic absorption edge shifts to the long-wavelength region with increasing Tm^3+^ concentration. Spectroscopic study of SMO:Tm^3+^/Ho^3+^ crystals [52] revealed that the maximum absorption cross-section of Tm^3+^ ions is observed at 795 nm and reaches 4.95 × 10^−20^ cm^2^ for σ-polarized radiation (*E* ⊥ *C*), which is higher than that in Y_3_Al_5_O_12_:Tm^3+^ and LiYF_4_:Tm^3+^ crystals. Thus, the most defective SMO:Tm^3+^ crystal with a disordered structure seems to be a promising material for the efficient radiation conversion in the region near 2-µm when pumped by a laser diode at 1700 nm.

#### 4.1.3. (Na,RE)*T*O_4_ (*T* = Mo, W; RE^3+^ = La, Gd)

The structural study of PbMoO_4_ and PbWO_4_ crystals (Section 4.1.1) showed that the most defective crystal is PbWO_4_, for which a decrease in symmetry was also observed. A similar situation occurs for complex scheelites with the general composition (Na_0.5_*RE*_0.5_)*T*O_4_ (*RE* = La, Gd; *T* = W, Mo) [17,61,107,109,136], however, the presence of several atoms in the dodecahedral site (Na^1+^ and *RE^3+^*) leads to some differences.

XRD analysis of both colorless and green (Na_0.5_Gd_0.5_)WO_3_ (NGW) crystals showed that ~ 4% and ~ 23% of diffraction peaks, respectively, do not obey the space group *I*4_1_*/a* [136]. Composition of the colorless NGW crystal, refined in the space group $I\bar{4}$ found also for PbWO_4_ crystals [104], can be written as [(Na_0.225(1)_Gd_0.275_)(1)(Na_0.229(1)_Gd_0.271_)(2)][(W_0.5_)(1)(W^6+^_0.458(2)_⎕_0.042_)(2)][(O_1.92(2)_⎕_0.08_)(1)O_2_(2)] (⎕-vacancies) with Gd > Na in both dodecahedral sites and vacancies in W(2) and O(1) sites (Cascales et al. [61] did not refine the site occupancies). It can be represented in the general form as (Na_0.454(1)_Gd_0.546_)(W^6+^_0.958(2)_⎕_0.042_)(O_3.92(2)_⎕_0.08_). A comparison of this composition with the composition of the same crystal refined for the subcell with the space group *I*4_1_/*a*, i.e., with fewer diffraction reflections–(Na_0.476_Gd_0.524(1)_)(W^6+^_0.992(2)_⎕_0.008_)O_4.00(1)_ [137], reveals common features: Gd > Na and vacancies in the W site. The differences are related to the compositions of O sites and formula coefficients for cations: 0 → Gd_Na_^m^^•^ + V_W_^n^′ + V_O_^p^^•^ (Equation (10)) or 0 → Gd_Na_^m^^•^ + V_W_^m^′ (Equation (11)) for the compositions refined in the framework of space group $I\bar{4}$ or *I*4_1_/*a*, respectively.

The composition of green NGW crystal can be written as [(Na_0.210(1)_Gd_0.290_)(1)(Na_0.290(1)_Gd_0.210_)(2)] [(W_0.5_)(1)(W_0.487(2)_⎕_0.013_)(2)] [(O_1.96(2)_⎕_0.04_)(1))O_2_(2)] (⎕-vacancies) with Gd > Na and Na > Gd in the first and second dodecahedral sites, respectively, with the total ratio Gd—Na. A comparison of this composition with the composition of the same crystal refined in the space group *I*4_1_/*a*–(Na_0.5_Gd_0.5_)(W_0.94(1)_⎕_0.06_)(O_3.82(6)_⎕_0.18_) [137], shows the same Gd and Na content with the same ratio Gd—Na in the dodecahedral site and the presence of W and O vacancies. The quasi-chemical reaction for the crystal with additional optical absorption is 0 → V_W_^n^′ + V_O_^m^^•^ + pe′ → V_W_^n^′ + V_O_^m^^•^ + W_W_^p^′ (Equation (12)). Thus, green color of NGW crystals is caused by a large number of O vacancies [138] and the associated free charge carriers (electrons) localized on W^6+^ ions: W^6+^ + e′ → W^5+^ [104,139].

Another structural picture is observed for crystals with the general composition (Na_0.5_Gd_0.5_)MoO_4_ (NGM) obtained at different growth and post-growth treatment atmospheres (Table 6).

The actual compositions of NGM-I and NGM-A crystals are (Na_0.493(3)_Gd_0.507_)Mo^6+^(O_3.920(9)_⎕_0.080_) and (Na_0.495(5)_Gd_0.505_)Mo^6+^(O_3.996(10)_⎕_0.004_) (⎕-vacancies), respectively, with approximately the same compositions of dodecahedral sites and different compositions of O sites (Table 6). Free (delocalized) charge carriers are responsible for the preservation of the electroneutrality and dark gray color of the NGM-I crystal (Table 6): 0 → Gd_Na_^n^^•^ + V_O_^m^^•^ + pe′ (Equation (13)). The slightly yellow color of the NGM-A crystal can be caused by the color center (V_Mo_^p^′,ph^•^)^×^ [139]. However, a low concentration of Mo vacancies in the NGM-A structure could not be fixed in this experiment. It should be noted that high vacancy content in the Mo site, which can be revealed by XRD analysis, is compensated by “holes” with the formation of color centers and leads to saffron-colored crystals [139].

The NGM-Ar is highly defective and has a significant variation in the composition over crystal volume. It is evidenced by different colors and different widths of diffraction peaks in different parts of the crystal: broad diffraction peaks (2.2°) for the NGM-Ar(1) compared with those (0.9°) for the NGM-Ar(2) (Table 6). The composition of the dark gray NGM-Ar(1) microcrystal (Na_0.489(4)_Gd_0.511_)(Mo_0.995(3)_⎕_0.005_)(O_3.915(10)_⎕_0.085_) (⎕-vacancies; 0 → Gd_Na_^m^^•^ + V_Mo_^n^′ + V_O_^p^^•^ + qe′; Equation (14)) differ from that of the almost colorless NGM-Ar(2) one (Na_0.498(2)_Gd_0.502_)(Mo_0.999(4)_⎕_0.001_)O_4_. As can be seen, the composition of the NGM-Ar(2) crystal is almost stoichiometric.

Thus, the compositions of NGM-I, NGM-A, and NGM-Ar crystals, grown in different atmospheres, differ from the composition of initial charge (Na_0.5_Gd_0.5_)MoO_4_: the Na content is less than the Gd one in the dodecahedral site (except for the NGM-Ar(2) composition with similar Na and Gd content). This is consistent with the rules of the polarity of isomorphism: (1) cations with smaller radii easily enter the crystal structure consisting of cations with larger radii; (2) for ions with similar ionic radii, the ion with smaller charge is easily replaced by the ion with higher charge [4]. Annealing in the air (NGM-A) of the sample (NGM-I) obtained in a slightly oxidizing atmosphere reduces the content of O vacancies (Table 6). Crystal growth in an Ar atmosphere leads to significant heterogeneity of crystal composition: defective and defect-free regions are observed for NGM-Ar. All the NGM crystals under investigation crystallize in the space group *I*4_1_/*a*.

The use of the non-stoichiometric composition of the initial charge with the lack of total (Na^+^ + Gd^3+^) amount in the dodecahedral site resulted in the growth of defective yellow-colored (Na_0.348(8)_Gd_0.528_⎕_0.124_)(Mo^6+^_0.996(3)_⎕_0.004_)O_4_ (NGM-2/7) and dark gray (Na_0.300(8)_Gd_0.576_⎕_0.124_)Mo^6+^(O_3.880(10)_⎕_0.120_) (NGM-6/15) crystals (⎕-vacancies), which differ from the compositions of initial charges (Na_0.286_Gd_0.571_⎕_0.143_)MoO_4_ and (Na_0.400_Gd_0.533_⎕_0.067_)MoO_4_, respectively (Table 6) [108]. Defect formation is described by the quasi-chemical reactions 0 → Gd_Na_^m^^•^ + V_(Na,Gd)_^k′^ + (V_Mo_^p′^,ph^•^)^×^ (Equation (15)) with a color center (V_Mo_^p′^,ph^•^)^×^ for the NGM-2/7 and 0 → Gd_Na_^m^^•^ + V_(Na,Gd_)^k’^ + V_O_^n^^•^ + *n*e′ (Equation (16)) with free charge carriers for the NGM-6/15. Moreover, in the NGM-6/15 crystal, in comparison with all other crystals, the highest content of O vacancies compensated by free charge carriers was revealed, which is also confirmed by its dark gray color (Table 6).

Morozov et al. [63] reported a light-violet crystal with a cation-deficient composition and incommensurately modulated structure grown in a slightly oxidizing (oxygen-deficient) atmosphere using the same charge composition Na_2/7_Gd_4/7_MoO_4_, followed by annealing in an oxygen atmosphere. A modulation was evidenced by additional satellite peaks at small angles of the diffraction spectrum. Incommensurately modulated (3 + 2)D scheelite-like structure of crystals with the nominal composition Na_2/7_Gd_4/7_MoO_4_ (superspace group *I*4¯(*α−β*0,*βα*0)00 with two modulation vectors q_1_ ≈0.54a* + 0.81b* and q_2_ ≈-0.81a* + 0.54b*) was determined by the single-crystal XRD and transmission electron microscopy. The composition of the grown crystals is close to that of the initial charge, but the presence of O vacancies in the structure is possible [63]. The calorimetric study of modulated crystals with the nominal composition Na_2/7_Gd_4/7_MoO_4_ indicates a phase transition of order (ordered modulated structure) - disorder (probably a structure with the space group *I*4_1_*/a*) type at 847 ± 6 °C. The reasons for the light-violet color are not discussed by Morozov et al. [63] However, an analysis of a large number of scheelite family crystals [104,139] indicates the partial transition Mo^6+^ → Mo^5+^. A similar crystal composition (Na_2/7_Gd_4/7_⎕_1/7_)MoO_4_:Nd (⎕-vacancies), but with the space group *I*4_1_/*a*, is reported by Zhao et al. [140] as congruently melting one with vacancies in one of two dodecahedral sites. It follows that the use of charge with the nominal composition (Na_2/7_Gd_4/7_)MoO_4_ may result in the growth of both unmodulated and modulated (with ordered structure) or defect and defect-free crystals depending on the growth and post-growth conditions (Table 6).

Structural study of Yb^3+^doped NGM crystals with the general compositions (Na_0.5_Gd_0.5_)MoO_4_:3.0 wt.% Yb (NGM:3Yb) and (Na_0.5_Gd_0.5_)MoO_4_:10 wt.% Yb (NGM:10Yb) showed that the number of additional diffraction reflections, not obeying the space group *I*4_1_*/a*, is 1.5% and 50%, respectively [109]. According to Cascales et al. [61], an increase in the Yb^3+^ content (r_Yb_^VIII^ = 0.99 Å, r_Gd_^VIII^ = 1.053 Å [84]; Δr/r_min_ = (r_Gd_-r_Yb_)/r_Yb_ ~6%) in the NGW:Yb crystals leads to a greater deviation from the centrosymmetry for the crystal NGW:Yb compared to NGW. It is evidenced by an increase in the number of diffraction peaks that do not obey the extinction laws of space group *I*4_1_*/a.* For NGW:10% Yb crystals ~50% additional peaks were revealed, while for NGW:10% Tm (r_Gd_^VIII^ > r_Tm_^VIII^ > r_Yb_^VIII^) ~4% additional peaks was found [141].

Refinement of the NGM:3Yb crystal structure in the space group *I*4_1_*/a* showed a presence of O vacancies and an absence of vacancies in the Mo site: (Na_0.500(1)_Gd_0.470(1)_Yb_0.030(2)_)Mo^5.94+^(O_3.970(5)_⎕_0.030_), which is described by the quasi-chemical reaction 0 → Mo_Mo_^×^ + V_O_^m^^•^ + pe′ → Mo_Mo_′ + V_O_^•^ (Equation (17)). The oxygen-deficient (i.e., insufficiently oxidizing) growth atmosphere promotes a partial reduction of Mo in the NGM:3Yb crystal (Equation (17)). The post-growth annealing in air leads to a decrease in the content of O vacancies and an increase in the formal charge (FC) of Mo: (Na_0.497(1)_Gd_0.471(1)_Yb_0.032(2)_)Mo^5.97+^(O_3.990(5)_⎕_0.010_) (⎕-vacancies).

An analysis of additional diffraction reflections in the NGM:10Yb microcrystal indicates a decrease in the symmetry to the space group $P\bar{4}$$P\bar{4}$, according to the reflections *hkl* with *h,k,l*–2*n* + 1, 00*l* with *l* = 2*n*. This space group was found for scheelite family crystals for the first time. For similar NGW:10Yb crystals, the revealed additional reflections belong to the space group $I\bar{4}$ [61,136]. Refinement of the NGM:10Yb crystal structure (XRD) within the subcell of space group *I*4_1_*/a* showed a wrong high deficiency of O site: Na_0.469(1)_Gd_0.479(1)_Yb_0.052(1)_Mo(O_2.0_⎕_2.0_) (⎕-vacancies). Pronounced peaks with coordinates 0.255, 0.598, −0.041; 0.245, 0.405, −0.041; 0.245, 0.405, 0.041 were observed in the residual electron density. These peaks are additional to the main O peak with coordinates 0.2579, 0.6022, 0.0406, found for NGM:10Yb, and increase the oxygen content in the phase up to real. In the transition from space group *I*4_1_*/a* to $P\bar{4}$, the crystallographic sites of atoms are split: 4*a* for (Na,Gd,Yb), 4*b* for W and 16*f* for O (space group *I*4_1_*/a*) are split into 1*a*, 1*d*, 2*g*^I^ for (Na,Gd,Yb)(1), (Na,Gd,Yb)(2), (Na,Gd,Yb)(3), 1*b*, 1*c*, 2*g*^II^ sites for Mo1, Mo2, Mo3 and 4*h*^I^, 4*h*^II^, 4*h*^III^,4*h*^IV^ sites for O(1), O(2), O(3), O(4) (space group $P\bar{4}$), respectively. The refined composition of the NGM:10Yb microcrystal can be written as [(Na_0.504(2)_Gd_0.470_Yb_0.026_)(1)(Na_0.510(2)_Gd_0.472_Yb_0.018_)(2)(Na_0.513(2)_Gd_0.472_Yb_0.014_)(3)][Mo(1)Mo(2)Mo(3)] [O(1),O_0.991(5)_(2),O(3),O(4)] or (Na_0.509(2)_Gd_0.471_Yb_0.020_)Mo^6+^(O_3.991(5)_⎕_0.009_) (in general form; ⎕-vacancies).

**Figure 3:**
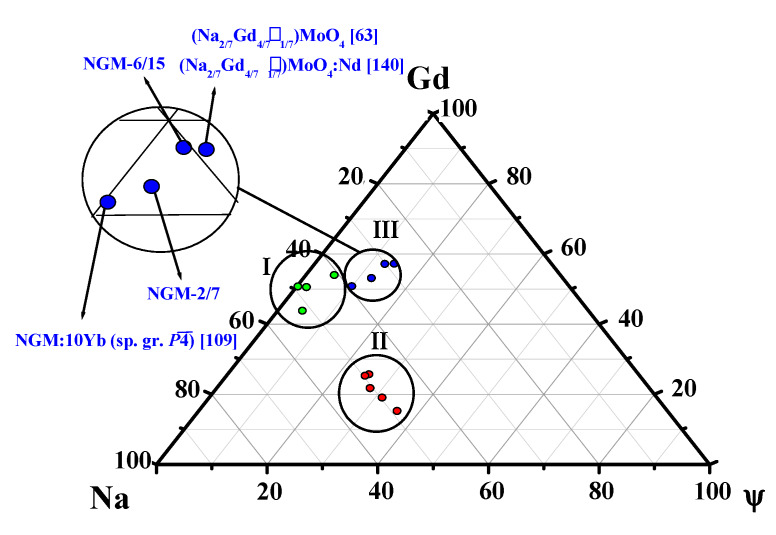
Region of existence of phases with the general composition (Na,Gd,ψ)MoO_4_ with ψ = 0, ⎕ (vacancy), Yb, La. Region III with defective and ordered structures is highlighted.

Figure 3 shows a triangle describing three regions of existence of phases with the general composition (Na,Gd,ψ)MoO_4_. Region I includes NGM-I, NGM-A, NGM-Ar phases [108] and phases with ψ = Yb (NGM:5.0Yb, NGM:1.5Yb, NGM:1.8Yb and NGM:4.0Yb [17]). Region II includes phases with ψ = La [17,142] with the space group *I*4_1_/*a*. Region III includes the defective phases with ψ = ⎕ (⎕–vacancies; phases with modulated structure [63] or without modulation [140] as well as phases NGM-2/7 and NGM-6/15 with the space group *I*4_1_/*a* [108]) and phase NGM:10Yb with the space group $P\bar{4}$ [109]. The presence of La^3+^ ions, having ionic radius higher than that of Gd^3+^ ion (r_La_^VIII^ = 1.16 Å, r_Gd_^VIII^ = 1.053 Å [84]) and comparable to that of Na^+^ ion (r_Na_^VIII^ = 1.18 Å [84]), in the dodecahedral site, prevents the ordering of atoms in this site. An increase in the Yb^3+^ content (r_Yb_^VIII^ = 0.99 Å [84]) in the initial charge composition up to 10% contributes to ordering [137]. The presence of vacancies in the dodecahedral site leads to the formation of ordered structures of different types provided that the integer (rational) formula coefficients of components in the dodecahedral site of the NGM structure (in particular, 0.286:0.571:0.143–2:4:1 for the phase (Na_0.286_Gd_0.571_⎕_0.143_)MoO_4_) (⎕-vacancies) are complied. It is possible that the disordered structures with vacancies in the dodecahedral site are formed in cases of a significant deviation of formula coefficients from integers (in particular, 0.348:0.528:0.124–2.81:4.26:1.00 for the phase (Na_0.348(8)_Gd_0.528_⎕_0.124_)(Mo^6+^_0.996(3)_⎕_0.004_)O_4_).

A reduction of symmetry observed in the local regions of scheelite family crystals indicates a kinetic phase transition of the order–disorder type (see, for example, [143,144]). In this case, a partially ordered (an ordered arrangement of different atoms over the corresponding crystallographic sites) noncentrosymmetric phase with the space group $I\bar{4}$ or $P\bar{4}$ is formed in the area of stability of the disordered (a statistical arrangement of several atoms over the crystallographic sites) centrosymmetric phase with the space group *I*4_1_/*a*. Analysis of summarized results of XRD studies of scheelite crystals together with their synthesis conditions allows us to distinguish two main determinants, i.e., structure and growth, which are responsible for the appearance of this phenomenon [109]. A key condition for such an ordering is, first of all, the presence of crystallographic sites jointly occupied by several atoms or atoms/vacancies, occurred in dodecahedral and tetrahedral sites of scheelite structures, respectively.

An ordering of atoms in phases with the scheelite structure depends not only on the matrix composition, type of activator ions and their concentrations, structural features, chemical and crystal–chemical properties of the components but also on the synthesis conditions, in particular, growth and post-growth atmospheres, crystallization, cooling and annealing rates, annealing temperatures, etc. The growth atmosphere affects the composition and hence the degree of atomic ordering in NGW crystals. For NGW:10Yb crystals grown in N_2_ and N_2_+O_2_ atmospheres, ~52% and 67% reflections, respectively, can not be indexed in the centrosymmetric space group *I*4_1_*/a* [137]. An increase in the rhombic (pseudotetragonal) distortion in the NGW:20Yb superstructure (*a ~*2*a_0_*, *c* ~2*c_0_*) of the scheelite structure (*a_0_*,*c_0_*) was found [137]. An increase in the growth rate of green and colorless NGW crystals from 4 to 6 mm h^−1^, respectively, decreases the degree of ordering [17]. The cooling rate of crystals has the same effect: a decrease in the cooling rate of NGW and NGW:Yb promotes the growth of ordered noncentrosymmetric crystals [61]. In addition, the degree of ordering decreases with an increasing number of annealing of NGW crystals [136].

Growth dissymmetrization is most pronounced under conditions close to equilibrium. Therefore, the symmetry of scheelite family crystals, and hence their properties, can be changed by varying the growth conditions, including the composition of initial charge as well as growth, cooling and post-growth treatment regimes.

The observed decrease in the symmetry of scheelite family crystals [17,61,107,136,145,146] explains crystal–chemical phenomena incompatible with the centrosymmetric space group. In particular, enantiomorphism was found for (Na_0.5_La_0.5_)MoO_4_ crystals [17,107] and the formation of racemic twins was revealed for the crystal with the nominal composition (Na_0.5_Gd_0.45_Yb_0.05_)MoO_4_ [17]. It should be noted that similar anomalous crystal–chemical phenomena and a presence of several different cations with different crystal–chemical properties (size, electronegativity, formal charge) in the same crystallographic sites should indicate possible kinetic phase transitions of order–disorder type, accompanied by a change in the symmetry and/or unit cell parameters. In this case, the ordering can be accompanied by the superstructure formation, but the disymmetrization of crystal structure found for the studied compounds due to kinetic ordering does not lead to a change in the structural type, at least, in the subcell of a specific structural type.

The disordering of dodecahedral sites in the scheelite structure, caused by the statistical distribution of Na^1+^ and *RE*^3+^ ions, broadens the absorption and photoluminescence spectral lines due to the inhomogeneity of the crystal field, which increases the efficiency of the radiative processes in comparison with compounds with ordered dodecahedral sites. However, the formation of superstructures (ordered phases) and defective phases depending on the growth conditions or the presence of controlled or uncontrolled impurities in the crystal composition should lead to the formation of distorted scheelite family structures, which will affect the structurally dependent properties.

Therefore, it is necessary to know the possible reasons for the appearance of such structural effects. As a result, the local coordination environment of atoms (XAS method) was studied in crystals with the nominal compositions (Na_0.5_Gd_0.5_)MoO_4_ (NGM 1:1) and (Na_2/7_Gd_4/7_⎕_1/7_)MoO_4_ (NGM 1:2) (⎕-vacancies) and refined compositions (XRD) (Na_0.498(2)_Gd_0.502_)(Mo_0,999(4)_⎕_0.001_)O_4_ (NGM-Ar(2)) and (Na_0.348(8)_Gd_0.528_⎕_0.124_)(Mo_0.996(3)_⎕_0.004_)O_4_ (NGM-2/7), respectively (Table 6). The CaMoO_4_ [14], having the same scheelite structure (space group *I*4_1_/*a*) as NGM 1:1 and NGM 1:2 crystals, is used here as a basis for comparison. One Ca^2+^ cation is located in the dodecahedral site of CaMoO_4_, in contrast to the NGM structure, where this site is occupied by two, Na^1+^ and Gd^3+^, cations.

Table 7 shows the cation–anion interatomic distances (*d*, Å) and the coordination spheres around the Ca^2+^ and Mo^6+^ cations (united by braces) in the CaMoO_4_ [14] structure.

Alternation of the coordination environment around the Ca^2+^ and Mo^6+^ cations (according to the structural analysis [14]) in the CaMoO_4_ crystal structure is the following (Table 7):

For Ca^2+^: the dodecahedron (4O1, 4O2), the tetrahedron (4Mo1), the tetrahedron (4O3), the dodecahedron (4Ca + 4Mo2). Dodecahedron with triangular faces (symmetry *S*_4_) is a tetragonal antiprism with refracted upper and lower faces;

For Mo^6+^: the tetrahedron (4O1), the tetrahedron (4O2) (such a sequence of negatively charged O^2−^ ions around the Ca^2+^ cation makes it possible to classify scheelite as quasi-layered structure), a polyhedron with 12 vertices (4Ca1, 4Ca2 + 4Mo). The polyhedron with 12 vertices (symmetry *S*_4_) is a dodecahedron with upper and lowers triangular and side quadrangular faces, i.e., tetragonal antiprism with refracted upper and lower faces and refracted two, upper and lower, opposite side edges.

If “complex cations” (Na^1+^,Gd^3+^) in the NGM structure statistically occupy Ca^2+^ sites in the scheelite CaMoO_4_ structure, a similar cationic coordination environment should be observed, i.e., “cations” (Na^1+^,Gd^3+^) should occupy Ca site (or Ca1 and Ca2 sites, which differ in interatomic distances) (Table 2).

**Figure 4:**
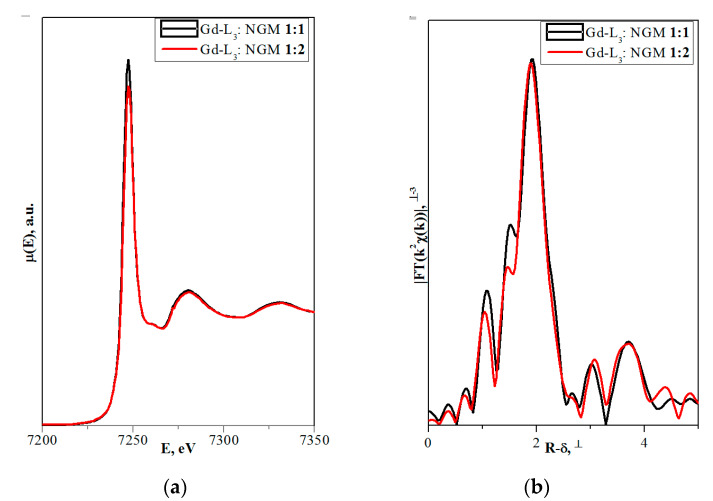
Gd L_3_-edge-normalized (**a**) XANES spectra and (**b**) EXAFS Fourier transforms for (Na_0.5_Gd_0.5_)MoO_4_ (NGM) 1:1 (black curves) and NGM 1:2 (red curves) samples.

**Figure 5:**
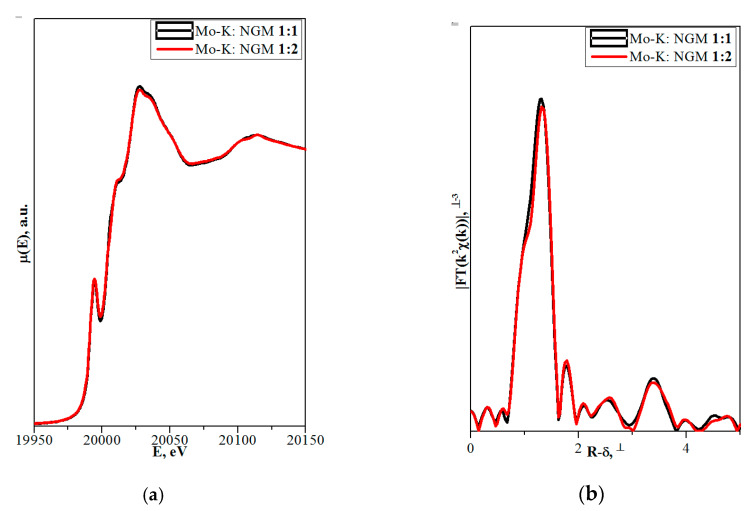
Mo K-edge-normalized (**a**) XANES spectra and (**b**) EXAFS Fourier transforms for NGM 1:1 (black curves) and NGM 1:2 (red curves) samples.

XANES spectra of NGM 1:1 and NGM 1:2 for both Mo K-edge and Gd L_3_-edge are nearly identical (Figure 4a and Figure 5a).

The results of EXAFS modeling are shown in Table 7. The most pronounced difference observed by XAS is a decrease in the Gd–Gd coordination number for NGM 1:2 compared to NGM 1:1 (Figure 4b). In the case of Mo K-edge, for the NGM 1:2 sample (Figure 5b), Mo–Na coordination numbers are decreased, while Mo–Gd coordination numbers are increased if compared to the NGM 1:1 (Table 7).

A comparison of the NGM and CaMoO_4_ structures (Table 7) shows that in the NGM, the Na^1+^ and Gd^3+^ ions are located at different interatomic distances from the Mo^6+^ ions rather than at the similar interatomic distances as in the case of the statistical arrangement of Na^1+^ and Gd^3+^ ions over the crystallographic site. At the same time, differences were found between the coordination environments (coordination spheres) around Mo^6+^ cations in the NGM 1:1 and NGM 1:2 structures while maintaining the sequence of alternating polyhedra as in the CaMoO_4_ structure (in Table 7, it is marked with an asterisk): the tetrahedron (4O1), the tetrahedron (4O2), a polyhedron with 12 vertices (2Na1, 2Gd1, 2Gd2, 4Mo, 2Na2) for the NGM 1:1 and the tetrahedron (4O1), the tetrahedron (4O2), a polyhedron with 12 vertices (1.2Na1, 2.8Gd1, 4Mo, 2.8Gd2, 1.2Na2) for the NGM 1:2.

Unfortunately, it was impossible to correctly determine the parameters of local structure from Gd L_3_-edge EXAFS due to large standard deviations. Therefore, the coordination environment of the Gd atom in NGM was considered to be similar to the Ca atom in CaMoO_4_ (Table 7). Nevertheless, according to the XAS method, vacancies in the Gd site, which were not detected in NGM 1:1 by XRD (Table 6), are possible in both NGM 1:1 and NGM 1:2 structures. Their content is greater in the NGM 1:2 structure, while the content of Na^1+^ ions is lower than that of Gd^3+^ ions in the NGM 1:2 structure compared to the NGM 1:1 one (corresponds to the results of XRD [108], Table 6). The total local composition of the NGM 1:1 can be estimated as “(Na^1+^_0.40_Gd^3+^_0.53_⎕_0.07_)Mo^6+^O_4_” (⎕-vacancies). The Mo–Gd and Mo–Na interatomic distances in NGM 1:1 and NGM 1:2—as well as the compositions of the coordination spheres around the Mo^6+^ ions—differ (Table 7). Thus, the Na^1+^ and Gd^3+^ ions form different coordination polyhedra with a different set of atoms surrounding them and with different interatomic distances. From Table 7 it follows that the disordering of atoms in NGM structures is observed in the third coordination sphere around the Mo^6+^ ion. It is likely that different local environments of Na^1+^ and *RE*^3+^, which can lead to a local change in the symmetry, should be expected in other crystals with scheelite structure having the general composition (Na^1+^,*RE*^3*+*^)*T*O_4_, caused primarily by the difference in the Na^1+^ and *RE*^3+^ formal charges.

The local structures (at the La/Eu L_3_-edge) of a series of samples with the general compositions (Na,La,Ca)MoO_4_ and (Na,Eu,Ca)MoO_4_, including the phases (Na_0.5_La_0.5_)MoO_4_ (NLM) and (Na_0.5_Eu_0.5_)MoO_4_ (NEM), were studied in a wide temperature range (15, 70, 180, 290 K) [147]. The coordination number for *RE* cations (*RE* = La, Eu) was only found to be 8 and the remaining coordination numbers were fixed or reached the limit set. (It should be noted that we did not fix the coordination environment for the Gd atom). Differences, occurred in structures when Ca is substituted for the La/Na or Eu/Na, are also given: increasing the amount of Eu increases the static disorder and the La-substitution series shows generally a much larger overall disorder. The size difference between La, Na and Ca introduces static perturbations of the local order already at low La concentration.

However, depending on the crystal–chemical properties of Na^1+^, *RE*^3+^ and *T*^6+^ ions (radii are the geometric factor and electronegativities are the chemical bond factor) as well as Na^1+^: *RE*^3+^ ratios and vacancies in the dodecahedral and tetrahedral sites in the (Na_0.5_*RE*_0.5_)*T*O_4_ structures, the symmetry of statistical structure determined by structural analysis may change. Due to the fact that the NLM and NGM crystallize in the space group *I*4_1_*/a* (according to XRD), the different Na^1+^ and *RE*^3+^ = La, Gd coordination polyhedra are distributed statistically, which does not lead to a change in the symmetry of the crystals. In the NGW structures, the Na^1+^ and Gd^3+^ polyhedra are arranged orderly, which is accompanied by a decrease in the symmetry from the space group *I*4_1_*/a* to $I\bar{4}$ This suggests that the local structure determined by the XAS spectroscopy is the root cause of further structural changes detected by diffraction methods.

It should be noted that it is relatively easy for the scheelite family compounds to change their symmetry (group–subgroup). Moreover, a significant role in the organization of the local structure of scheelite compounds belongs to isomorphism factors. Therefore, it is necessary to perform a structural experiment on single-crystal objects with a careful analysis of diffraction peaks, including weak ones. The use of high-resolution transmission electron microscopy and synchrotron radiation for this purpose is also encouraged. It is advisable to additionally use X-ray absorption spectroscopy at different absorption edges (preferably, at low temperatures), followed by a joint analysis of the structural results. The final important stage is a choice of optimal software and the development of the strategy of refining the site occupancies in the structure, i.e., determination of the actual composition in a real structure. This is highly important for: (1) the correct determination of correlations between symmetry, actual composition of samples and growth conditions; (2) the proper explanation the observed functional properties; (3) the directed growth of crystals with the desired combination of characteristics; (4) the clarification and summarization of crystal–chemical knowledge using the scheelite family compounds as an example.

In conclusion, it should be noted that the color of the scheelite family crystals grown by the Czochralski method can be caused by various factors: (1) the presence of activator ions (Tm—green, Er—pink, Ce—yellow, Yb—colorless); (2) the formal charge of Mo (Mo^5+^-lilac), W (W^5+^-green) and Pb (Pb^3+^-yellow) ions; (3) the presence of oxygen vacancies with delocalized (dark gray or black) charge carriers [139] or oxygen or cation vacancies with localized charge carriers with the formation of color centers. According to Suvorova et al. [148], the opaque crystals are formed due to the CeO_2_ precipitates occurred on the inner surfaces of NLM:Ce,Er and, probably, *RE*_2_O_3_ in the NLGM:Tm, appeared as a result of post-growth treatment of the samples in the air at high temperatures (*T* = 1000 °C). This process is facilitated by the structural-geometric correspondence between scheelite and *RE* structures. Knowing the reasons for this phenomenon made it possible to obtain scheelite family NLM:Ce,Er crystals of high optical quality as a result of continuous post-growth annealing (τ = 100 h) at relatively low temperature (*T* = 700 °C).

### 4.2. Eulytin Family Compound Bi_4_Ge_3_O_12_

**Figure 6:**
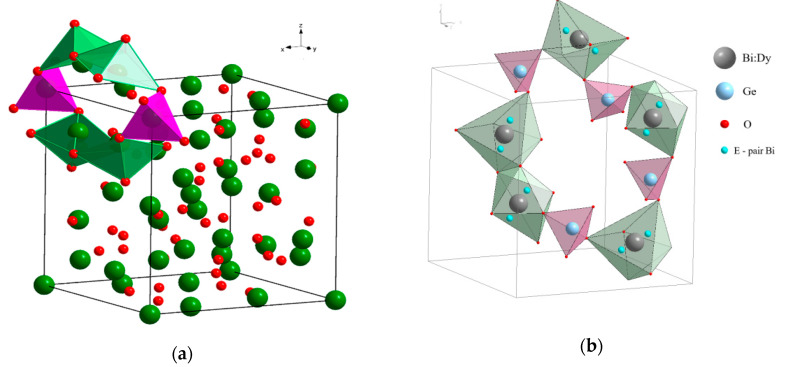
Coordination polyhedra in the (**a**) Bi_24_*M*_2_O_40_ and (**b**) Bi_4_Ge_3_O_12_ structures.

Among the oxides of complex compositions, a special place is occupied by the compounds containing bismuth ions, which may have different formal charges (FC). The presence of an active or passive lone electron pair (*E*-pair) is typical for Bi^3+^. It is these chemical features that lead to the realization of structural characteristics inherent only to these compounds. The sillenite family compounds and solid solutions with the general compositions Bi_24_*M*_2_O_40_ or Bi_24_(*M′_,_M″*)_2_O_40_ (space group *I*23; Z = 1) (Figure 6a) [149] can be given as an example. For these materials, a special methodology was developed to refine their actual compositions and structures [150], taking into account the FC Bi and the distribution of Bi^3+^ and Bi^5+^ over different crystallographic sites [151]. For this family of compounds, the use of structural analysis in combination with X-ray absorption spectroscopy has been successful. As a result, this methodology was successfully applied to another class of compounds with the eulytin structure [110].

**Figure 7:**
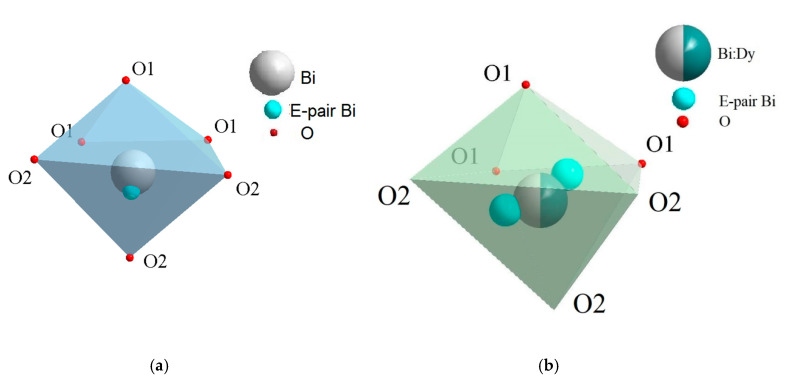
(**a**) BiO_6_ polyhedron with a lone electron pair in the Bi_4_Ge_3_O_12_ structure; (**b**) The (Bi,Dy)O_6_ polyhedron in the BGO:Dy structure. Oxygen atoms distributed over one right system of points in the BGO structure are conventionally marked as O1 and O2.

Bismuth orthogermanate Bi_4_Ge_3_O_12_ (BGO) crystallizes in the eulytin Bi_4_(SiO_4_)_3_ structure (space group$\;I\bar{4}3d$, *Z* = 4) [152]. The Bi^3+^ cations occupy the Wyckoff site 16*c* with coordinates *x x x* forming the distorted BiO_6_ octahedra with two different Bi–O interatomic distances with CN = 3 + 3 due to the presence of an active lone electron pair (*E*-pair) (Figure 6b and Figure 7a). The Ge^4+^ cations occupy the Wyckoff site 12*a* with coordinates 3/8 0 1/4 and form the tetrahedra with four equal Ge–O distances (CN Ge = 4). The O^2−^ ions are coordinated, in turn, by two Bi atoms and one Si atom and occupy general site 48*e* with coordinates *x y z*.

In the BGO crystal structure, the Bi^3+^ and Ge^4+^ ions are located, respectively, on the 3 and $\bar{4}$ axes. The distorted BiO_6_ octahedron is connected with the GeO_4_ tetrahedra by the vertices and with another BiO_6_ polyhedron by the edge. GeO_4_ and BiO_6_ polyhedra form rings. The eulytin structure can be described as a skeleton, similarly to the sillenite structure.

According to XRD data, BGO crystals have different actual compositions depending on their color. Colorless BGO(C) and pink BGO(P) samples have the refined compositions (Bi^3+^_3.994(40)_⎕_0.006_)[Ge_2.980(30)_Bi^5+^_0.020_)O_12.00_ (0 → V_Bi_^n^′ + Bi_Ge_′ + Bi_Bi_^m•^; Equation (18)) and (Bi^3+^_3.987(45)_⎕_0.013_)(Ge_2.988(35)_Bi^5+^_0.012_)(O_11.98(5)_⎕_0.02_) (⎕-vacancies; 0 → V_Bi_′^m^ + Bi_Ge_′ + 2Bi_Bi_^•p^ + (V_O_^•n^, ne′)^×^; Equation (19)), respectively (Table 8). The pink color of the BGO(P) crystals is due to the color center (V_O_^•n^, ne′)^×^ (electrons localized on oxygen vacancies) due to the reducing growth atmosphere. It should be noted that the similar change in the color of garnet Gd_3_Ga_5_O_12_ crystal from colorless to pink was also caused by the presence of a color *F-*center (V_O_^••^,2e′)^×^ [153].

Bravo et al. [75], based on the decreased unit cell parameters of BGO:Nd^3+^ crystals compared to those of undoped BGO (r_Bi_^VI^ = 1.03 Å, r_Nd_^VI^ = 0.98 Å [84], Δr/r_min_ = (r_Bi_^VI^-r_Nd_^VI^)/r_Nd_^VI^ ~5%; r_i_ is the ionic radius of ions with the CN = 6), concluded that the Nd^3+^ ions isomorphically replace Bi^3+^ ions. According to the isomorphism theory, in addition to the geometric factor, the electronegativity factor should also be taken into account: electronegativity values are different for Bi and Nd χ_Bi_ = 1.8, χ_Nd_ = 1.2, Δχ = 0.6). It should be noted that in the eulytin structure, the Bi^3+^ ions have an active electron pair, which should also prevent isomorphic substitution by an ion without an *E* pair. All these crystal–chemical factors will affect the structural features of doped crystals of the eulytin family, for which any single-crystal structural studies with further refinement of occupancies of crystallographic sites have not been previously performed.

Analysis of diffraction reflections of BGO:Dy^3+^ microcrystals (XRD) indicates that for the unit cell with the BGO parameters, about 13% of reflections do not obey the space group $I\bar{4}3d$. As a result, a real symmetry of the crystals is to be lower (it should be noted that only 5% of additional reflections were observed for nominally pure BGO crystal [110]). A similar was established for the sillenite-family crystals with the general formula Bi_24_*M_2_*O_40_: a symmetry reduction from the space group *I*23 to *P*23 is caused by the presence of atoms with different crystal–chemical properties in one crystallographic *M* site as well as by the growth factors [149,154]. Similar effect is described for the scheelite family crystals in Section 4.1.

Nevertheless, the refinement of the BGO:Dy structures was carried out within the framework of the space group $I\bar{4}3d$ due to the relatively small quantity of additional reflections and a sharp increase in the number of refined parameters in the case of lower symmetry, which results in instable calculations and incorrect structural parameters [7,155]. The actual composition of the BGO:1.0Dy crystal can be written as (Bi_3.952(47)_Dy_0.048_)Ge_3_O_12_ with a partial replacement of Bi^3+^ ions by the Dy^3+^ ones: 0 → Dy_Bi_^×^ (Equation (20)) (Table 8).

A refinement of BGO:1.0Dy structure revealed residual electron density (*F_o_*–*F_c_*) peaks with heights of 3.41 and 3.10 electron units and coordinates 0.0893 0.0831 0.1571 and 0.0939 0.0870 0.0189, respectively. The coordinates of Bi atom (*x x x*) in the BGO:1.0Dy inverted structure are 0.08741 0.08741 0.08741, i.e., a residual electron density is located on both sides of the Bi site (Figure 7b).

A concentration of Dy^3+^ ions in the BGO:0.1Dy crystal cannot be refined correctly due to the large standard deviation, therefore, the most probable composition is (Bi^3+^_3.996(11)_Dy_0.004_)Ge_3_O_12_ (Table 8). Comparison of the results of structural analysis of BGO:0.1Dy and BGO:1.0Dy samples indicates that an increase in the Dy content leads to a decrease in the residual electron density. It seems quite logical assuming a responsibility of the active electron pair of Bi^3+^ for the picture observed and a decrease in the height of the residual electron density peak with increasing content of Dy^3+^ ions in the structure without an active *E*-pair. Compared to undoped BGO crystals, BGO:Dy crystals are less defective and the Bi^5+^ ions are absent in the tetrahedral site (Table 8), as was also observed for sillenite family crystals [151].

A partial presence of Dy^3+^ ions in the Bi site of BGO:Dy crystal structure contributes to the symmetrization of the octahedron, i.e., to an increase in the Bi–O1 distance. Indeed, according to the calculation of the degree of distortion (δ) of the BiO_6_ polyhedron using the formula δ = Σ∆*d_i_*^2^/(CN–1) (Equation (21); Δ*d* is a difference in distances between the vertices of a distorted and ideal coordination polyhedron; the *i* is changed from 1 to CN), its value in the structure of BGO:1.0Dy crystal (δ = 0.02) is found to be lower than that in the nominally pure BGO structure (δ = 1.855). This is due to the presence of the activator ion in the BGO:1.0Dy structure, which reduces the effect of an active *E*-pair.

**Figure 8:**
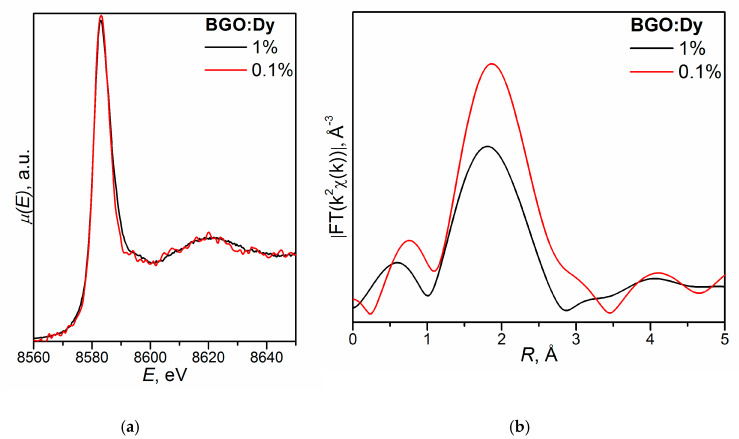
Dy L_2_-edge (**a**) XANES spectra and (**b**) EXAFS Fourier transforms (*k*-range, 2–6 Å^−1^) for the BGO:1.0Dy and BGO:0.1Dy samples.

However, differences in the sizes of Bi^3+^ and Dy^3+^ ions (r_Bi_^VI^ = 1.03 Å; r_Dy_^VI^ = 0.91 Å [84]; Δr/r_min_ = (r_Bi_^VI^-r_Dy_^Vi^)/r_Dy_^VI^ ~13 and in their electronegativity values (χ_Bi3+_ = 1.8, χ_Dy3+_ = 1.2, Δχ = 0.6) should affect the local structure. One can see that XANES data for BGO:Dy samples is nearly identical, therefore, the formal charge of Dy in BGO:1.0Dy and BGO:0.1Dy is similar (Figure 8a). Moreover, XANES spectrum is very similar to the data for Dy_2_O_3_ which can be found elsewhere [156]. Therefore, in both samples, Dy ions have FC = 3.

EXAFS Fourier transforms, despite the low resolution, reveal a maximum at ~1.9 Å, which corresponds to Dy–O coordination (Figure 8b). Moreover, the intensity of Dy–O peak is substantially higher for BGO:0.1Dy compared to BGO:1.0Dy. Since no major differences in XANES are observed (i.e., the coordination type of Dy in both samples is mostly similar) and the difference in Dy–O peak intensity is nearly 50%, we suggest that this difference is mostly due to the lower Dy coordination number in the BGO:1.0Dy (the results of the single-sphere fit are shown in Table 9).

Available *k*-range is too small to allow the complicated fit involving multiple coordination spheres. However, the data indicate that single Bi–O distance is insufficient for the correct modeling of Dy local surroundings. If the upper limit of *k*-range is increased to 8, the Dy–O maximum is split into two smaller peaks, suggesting two different Dy–O distances.

**Figure 9:**
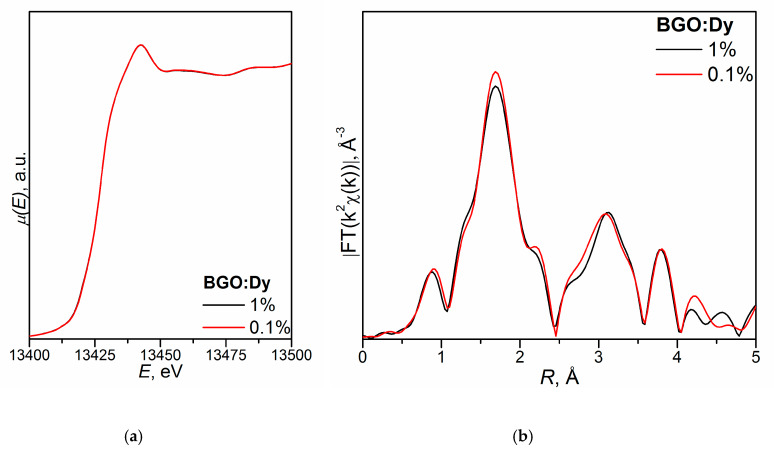
Bi L_3_-edge (**a**) XANES spectra and (**b**) EXAFS Fourier transforms (*k*-range, 2–13 Å^−1^) for the BGO:1.0Dy and BGO:0.1Dy samples.

The Bi L_3_-edge XANES and EXAFS spectra for both samples were also measured (Figure 9).

**Figure 10:**
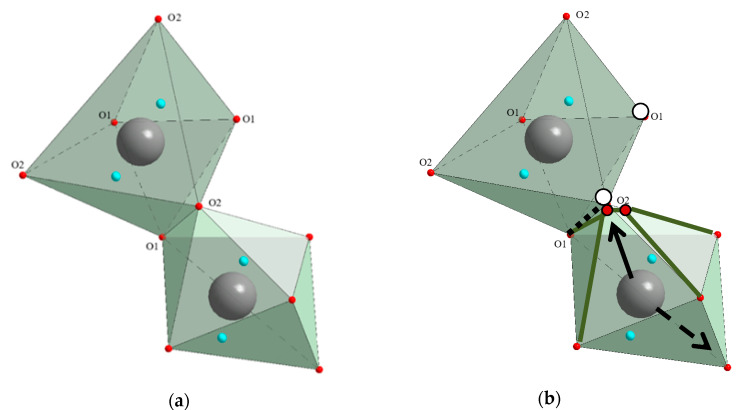
Model of rearrangement of (**a**) the BiO_6_ and DyO_6_ octahedra into (**b**) the vacancy BiO_5_ octahedron and DyO_7_ octahedron with a split top. The solid arrow shows the direction of Dy^3+^ displacement with the formation of the DyO_7_ polyhedron. The dashed arrow shows the direction of Dy^3+^ displacement o with the formation of the DyO_5_ polyhedron.

Table 9 shows that the CN Dy = 7 and CN Bi = 5 (2.5 + 2.5) in the BGO:0.1Dy structure and CN Dy = 5 and CN Bi = 6 (3 + 3) in the BGO:1.0Dy structure (Figure 10).

**Figure 11:**
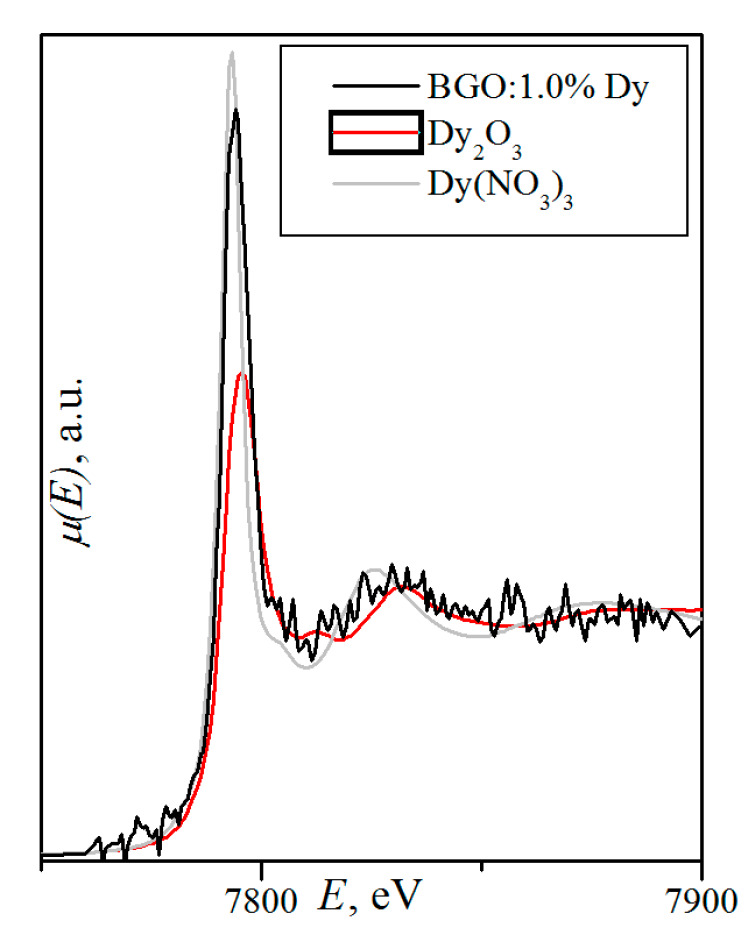
Dy L_3_-edge XANES data for the BGO:1.0Dy and reference samples: Dy_2_O_3_ and Dy(NO_3_)_3_.

A change in the Dy^3+^ coordination polyhedron in the BGO:1.0Dy structure follows from the Dy L_3_-edge XANES spectrum (Figure 11).

Although the spectrum is noisy, it can be seen that the shape of XANES is closer to that measured for the Dy(NO_3_)_3_, in the structure of which the Dy is located in a coordination polyhedron based on a trigonal prism, rather than octahedron as in the Dy_2_O_3_ structure. However, the positions and relative intensities of the main features are similar for all three spectra.

The possible quasi-chemical reaction in local single polyhedra in the BGO:0.1Dy can be described as 0 → Dy_Bi_^×^ + V_O_^p•^ + O_i_^q′^ (Equation (22)): V_O_^p•^–oxygen vacancies in the BiO_6_ octahedron → vacancy octahedron BiO_5_ (CN Bi = 2.5 + 2.5), O_i_^q^′^–^interstitial oxygen atoms → octahedron with a split top or distorted one-capped trigonal prism (CN Dy = 5 + 2) (Figure 10). The possible quasi-chemical reaction in local single polyhedra in the BGO:1.0Dy can be written as: 0 → V_Dy_^n^′ + Bi_i_^m•^ (Equation (23)): Dy^3+^ displacement to the top of the DyO_6_ octahedron → semi-octahedron or polyhedron derived from trigonal prism DyO_5_ (CN Dy = 5), octahedron BiO_6_ (CN Bi = 3 + 3).

In terms of XRD methods, the structural parameters of the sample are reduced to the parameters of a single unit cell, and the sample is considered as a set of identical cells. Some of the sites in one “universal” cell may, however, be partially vacant or simultaneously occupied by different chemical elements. Two partially occupied sites may even be at a distance less than the sum of the ionic radii of the corresponding atoms, which means the mutually exclusive occupation of one or another site in different copies of the “universal” cell. Thus, in terms of X-ray crystallography, all unit cells are equivalent.

In terms of XAS, which is an element-specific technique, this is not the case. The Dy L_2_-edge XAS provides us with information on the structure of Dy local surroundings. Therefore, XAS ignores “normal” copies of the BGO “universal” cell, which contains only Bi, Ge and O atoms and considers only “defective” copies that also contain Dy. More specifically, the Dy L_2_-edge XAS considers only “defective” polyhedra that contain Dy instead of Bi. Similarly, the Bi L_3_-edge XAS considers only “normal” polyhedra with Bi and ignores “defective” polyhedra with Dy. That’s why it is possible to build up different local structure models for Bi and Dy, even though, in terms of crystallography, these atoms are equivalent, as they occupy the same site.

It follows that the structural behavior of the Dy^3+^ activator ions depends on their content in the crystal, i.e., a “concentration effect” occurs. It leads to the formation of a local structure of different composition and structure (BiO_5_ and BiO_6_; DyO_5_ and DyO_7_) (EXAFS), which is consistent with the crystallochemical concepts as well as with a different degree of symmetrization of the statistical (Bi,Dy)O_6_ octahedron (XRD). As a result, the symmetry of polyhedra (symmetry *3* for BiO_x_, symmetry *1* for DyO_y_ according to EXAFS; symmetry *3* for (Bi,Dy)O_6_ according to XRD) and BGO:Dy structure is reduced taking into account ~13% additional reflections (XRD). It should be noted that a “concentration effect”, but of a different kind, was revealed for the Sr_0.61_Ba_0.39_Nb_2_O_6_:Ni crystals [157]: an increase in the Ni content in the initial batch from 0.5 wt% to 1.0 wt% results in a change in its formal charge from 3+ to 2+ in the grown crystal.

**Figure 12:**
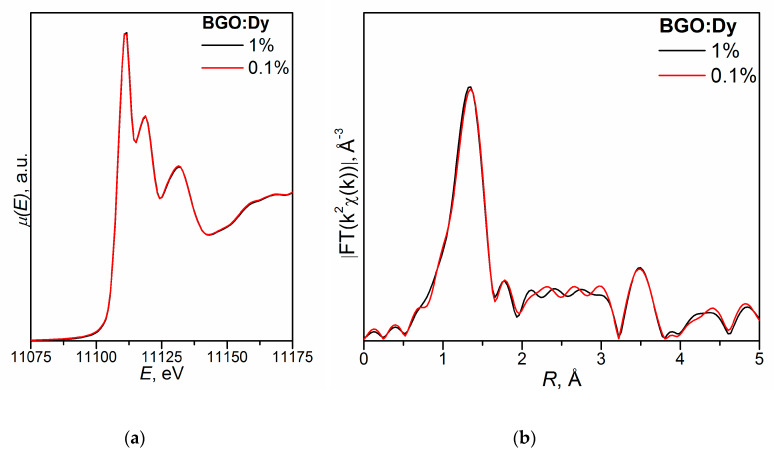
Ge K-edge (**a**) XANES spectra and (**b**) EXAFS Fourier transforms (*k*-range, 2–13 Å^−1^) for the BGO:1.0Dy and BGO:0.1Dy samples.

For Ge K-edge, no significant differences are observed (Figure 12) and the Ge^4+^ ions have a tetrahedral environment.

The results of the XRD and XAS investigations of BGO and BGO:Dy crystals [110,120] indicate that the introduction of more than 1.0 wt% Dy into the Bi^3+^_4_Ge_3_O_12_ crystal matrix leads to the formation of two-phase samples.

### 4.3. Perovskite Family Compound CsCdX_3_ (X = Cl, Br) as well as TlCdX_3_ (X = Cl, I)

Bismuth ions exhibit their specific features not only as the matrix ions, as was observed in the sillenite and eulytin family structures (Section 4.2), but also as activator ions.

#### 4.3.1. CsCd*X*_3_ (*X* = Cl, Br)

In the perovskite CaTiO_3_ structure (space group *Pm*3*m*, *Z* = 1), the Ca^2+^ cations occupy the Wyckoff site 1*b* with coordinates 0.5 0.5 0.5 forming the cuboctahedra with CN = 12 [158]. The Ti^4+^ cations occupy the Wyckoff site 1*a* with coordinates 0 0 0 and form the octahedra with CN Ti = 6. The O^2-^ ions occupy the Wyckoff site 3*c* with coordinates 0.5 0 0 and are coordinated by two Ti atoms and four Ca atoms (CN O = 6). The structure of perovskite can be described as a skeleton: the TiO_6_ octahedra are connected by tops and the Ca^2+^ ions having the dodecahedral environment are located in the skeleton cavities.

**Figure 13:**
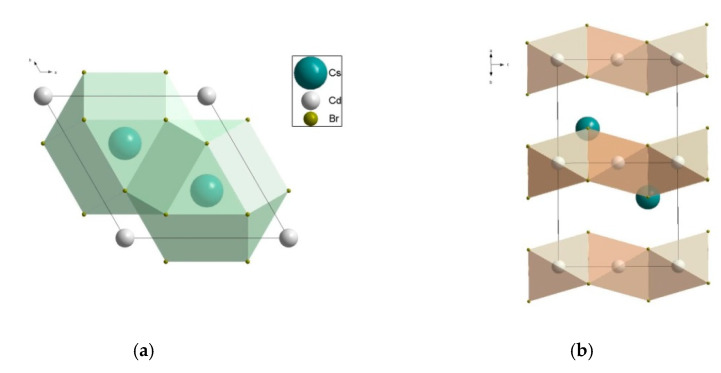
Crystal structure of CsCdBr_3_: (**a**) the *xy* projection with polyhedra for the Cs atoms; (**b**) the *xz* projection with polyhedra for the Cd atoms.

**Figure 14:**
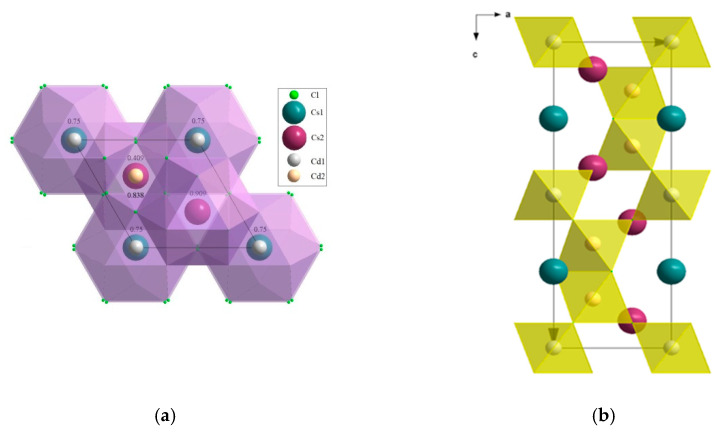
Crystal structure of CsCdCl_3_: (**a**) the *xy* projection with polyhedra for the Cs atoms; (**b**) the *xz* projection with polyhedra for the Cd atoms.

The CsCdBr_3_ and CsCdCl_3_ compounds belong to the perovskite family (*a*_0_ ~2(r_Cd_^VI^ + *R*_X_) Å, where *a*_0_ is a unit cell parameter of the cubic basic structure; r_Cd_^VI^ and *R*_X_ are ionic radii for the Cd^2+^ and *X* = Cl, Br [84]) and crystallize in the space group *P*6_3_*/mmc* with the unit cell parameters *a* = 7.675(3), *c* = 6.722(3) Å and *a* = 7.418(4), *c* = 18.39(3) Å, respectively [159,160]. In the CsCdBr_3_ (Figure 13) and CsCdCl_3_ (Figure 14) crystal structures, Cs and Cd atoms are located, respectively, in hexagonal cuboctahedra with two different interatomic distances Cs–Br (Cl) with CN = 6 + 6 and octahedra.

The CsCdBr_3_ crystal structure (CsNiCl_3_-type structure) can be described as a two-layer hexagonal packing (*h*-packing) of CsBr_3_ trigonal layers (Figure 13). The structure of CsCdCl_3_ (CsMgF_3_-type structure) can be represented as a mixed six-layer hexagonal packing (*hcc*-packing) of CsCl_3_ layers, the octahedral voids in this packing being completely occupied by the Cd^2+^ ions (Figure 14) [161].

An application of the theory of isomorphic miscibility of components [4], in particular, the geometric factor (r_Cs_^XII^ = 1.88 Å, r_Bi_^XII^ = 1.903 Å [90]; Δr/r_min_ = (r_Bi_–r_Cs_)/r_Cs_ = 1%; r_Cd_^VI^ = 0.95 Å, r_Bi_^VI^ = 1.03 Å [84]; Δr/r_min_ = (r_Bi_–r_Cd_)/r_Cd_ = 8%) indicates that the radii of Cs^1+^ and Bi^1+^ ions (in the case of the implementation of the Bi^1+^ ion) as well as those of Cd^2+^ and Bi^3+^ ions (in the case of the implementation of the Bi^3+^ ion) are similar. Hence, the substitution of Cs^1+^ ions by the Bi^1+^ ones (preferably according to size factor) or Cd^2+^ ions by the Bi^3+^ ones is possible. However, in addition to similar sizes of atoms, it is necessary to take into account their electronegativity values. From this point of view (χ_Cs_^1+^ = 0.7 and χ_Bi_^1+^ = 1.4, Δχ = 0.7; χ_Cd_^2+^ = 1.5 and χ_Bi_^3+^ = 1.8, Δχ = 0.3), the substitution of Cd^2+^ for the Bi^3+^ ions is most likely. The difference in the formal charges of the Cd^2+^ and Bi^3+^ should also be taken into account, as was applied for the Na^1+^ and *RE*^3+^ ions in the (Na_0.5_*RE*^3+^_0.5_)*T*O_4_ structures.

The refinement of the CsCdBr_3_ crystal structure based on the synchrotron data resulted in the stoichiometric composition. The refined composition of the CsCdBr_3_:Bi was found to be (Cs_0.976(10)_⎕_0.024_)(Cd_0.967(47)_Bi^3+^_0.033_)Br_3_ (⎕-vacancies; 0 → 2V_Cs_′ + Bi_Cd_^•^; Equation (24)). According to the results of the refinement of occupancies for all cationic sites in the CsCdCl_3_:Bi structure determined at 200 K (a slight decrease in the Cd2 site occupancy within the accuracy of determination is only observed), Bi ions did not enter the crystal structure of the sample under investigation. However, according to XRD study of another part of the same crystal, the refined composition is (Cs1_0.989(1)_⎕_0.011_)Cs2(Cd1_0.995(2)_Bi^3+^_0.005_)(Cd2_0.996(2)_Bi^3+^_0.004_)Cl_6_ (⎕-vacancies; 0 → 2V_Cs_′ + Bi_Cd_^•^; Equation (24)), which indicates: (1) the heterogeneity of the CsCdCl_3_:Bi crystal composition; (2) the greater deficiency of crystals containing Br^1−^ ions (*R*_Br_^VI^ = 1.96 Å [84]) compared to Cl^1−^ ions (*R*_Cl_^VI^ = 1.81 Å [84]); (3) the absence of monovalent Bi^1+^ ions.

**Figure 15:**
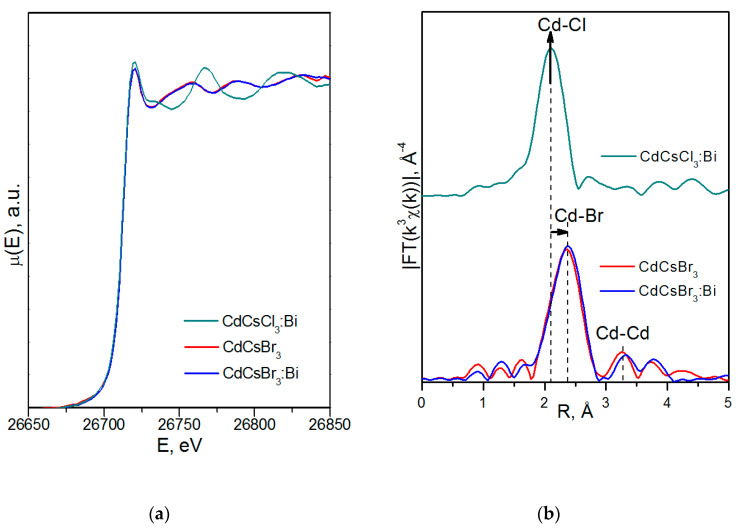
Cd K-edge (**a**) XANES spectra and (**b**) EXAFS Fourier transforms for the.CsCdBr_3_, CsCdBr_3_:Bi and CsCdCl_3_:Bi samples.

The XANES spectra measured at the Cd K-edge for the CsCdBr_3_ and CsCdBr_3_:Bi samples are identical (Figure 15a). The XANES spectrum for the CsCdCl_3_:Bi has a similar structure near the absorption edge, while more distant oscillations differ due to different local atomic structures of chloride and bromide.

The EXAFS technique is sensitive to a chemical element. However, atoms of the same element, located in different crystallographic sites, can not be distinguished (relevant for CsCdCl_3_) [118]. The EXAFS fitting procedure at the Cd K-edge for the CsCdCl_3_:Bi sample (Figure 15b and Table 10) results in the significantly increased Cd–Cl interatomic distance (*d*_EXAFS_ = 2.72 Å) compared with the structural data for the CsCdCl_3_ [159].

This means that there is an additional electron density at a distance of ~2.7–2.8 Å from the Cd atom. However, a presence of Bi atoms at or near the Cd site cannot be found based on the EXAFS data. Nevertheless, it is clear that the Bi^3+^ and Cd^2+^ ions form different (neighboring) polyhedra. It confirms that the numerical values of isomorphism factors are much smaller in the local approximation (XAS) than in the statistical one (XRD).

The EXAFS Fourier transform curves for the CsCdBr_3_ and CsCdBr_3_:Bi samples differ slightly: the intensities and positions for peaks of the first coordination sphere are identical (Figure 15b). However, for the second coordination sphere with a maximum at 3.3 Å occupied by the Cd atoms, a simultaneous increase in the Debye factor and a small (within the error of ±1) increase in the CN is observed for the CsCdBr_3_:Bi sample. It may be due to the substitution of Cd atoms for the heavier Bi ones (Bi_Cd_^•^) and the associated structural disorder. This conclusion does not contradict the structural data.

#### 4.3.2. TlCd*X*_3_ (*X* = Cl, I)

**Figure 16:**
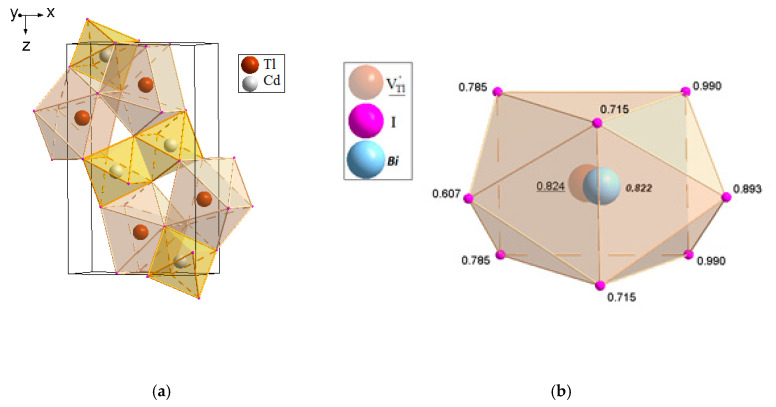

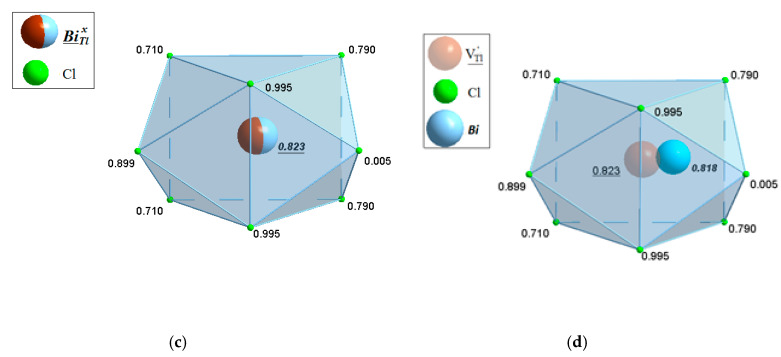
(**a**) Structure of TlCd*X*_3_ (*X* = Cl, I); (**b**) The (Tl,Bi_i_)I_8_, (**c**) (Tl,Bi)Cl_8_, (**d**) (Tl,Bi_i_)Cl_8_ coordination polyhedra.

The transition from the CsCd*X*_3_ (*X* = Cl, Br) to TlCd*X*_3_ (*X* = Cl, I) compounds should be accompanied by a change in symmetry (i.e., crystallization in different structural types), which follows from the first and second principles of crystal chemistry: r_Cs_^XII^ = 1.88, r_Tl_^XII^ = 1.70 Å [84], Δr/r_min_ = (r_Cs_^XII^-r_Tl_^XII^)/r_Tl_^XII^ ~11%, the size factor; χ_Tl_ = 1.5, χ_Cs_ = 0.75, Δχ = 0.75, the chemical bonding factor [7]. Indeed, the TlCdCl_3_ and TlCdI_3_ are isostructural (and not isostructural to CsCdX_3_) and crystallize in the space group Pnma (Z = 4) (Figure 16) [162,163].

In the TlCd*X*_3_ crystal structure, the Cd atoms form distorted octahedra with different interatomic distances (CN Cd = 2 + 2 + 1 + 1), and the Tl atoms are in distorted bicapped trigonal prisms (CN Tl = 2 + 2 + 2 + 1 + 1) (Figure 16). The Cd*X*_6_ polyhedra as well as the Tl*X*_8_ ones are combined *via* edges to form chains along the <110> direction. The Cd*X*_6_ and Tl*X*_8_ polyhedra are connected by edges along the <111> direction (Figure 16).

The refinement of the Tl site occupancy in the TlCdI_3_:Bi structure showed its decrease, which excludes the additional presence of Bi ions in this site. The Cd and I sites found to be defect-free. In the residual electron density of TlCdI_3_:Bi sample, the peak with Δρ_max_ = 3.08 was detected. It can be responsible for the interstitial Bi^1+^ ions (Bi_i_^n•^) with the formal charge 1+ (according to the electroneutrality condition), located near the Tl^+^ ions (Figure 16b). Defect formation in the TlCdI_3_ crystal with the actual composition (Tl^1+^_0.980(12)_⎕_0.020_)Bi^1+^_(i)0.014(12)_CdI_3_ can be described by a quasi-chemical reaction: 0 → V_Tl_^n^′ + Bi_i_^n•^ (Equation (25)), i.e., Tl^1+^ ions (r_Tl_^VIII^ = 1.59 Å [84]) are not replaced by Bi^1+^ ions (r_Bi_^VIII^ = 1.774 Å [84]; Δr/r_min_ = (r_Bi_-r_Tl_)/r_Tl_ = 11%), as assumed in Refs [89,91,101,102,164,165,166,167].

The actual composition of the TlCdCl_3_:Bi crystal was found to be (Tl^1+^_0.998(1)_⎕_0.002_)Bi^1+^_(i)0.005(1)_CdCl_3_ with vacancies in the Tl site and interstitial Bi^1+^ ions: 0 → V_Tl_^n^′ + Bi_i_^n•^ (Equation 25) (Figure 16c). However, the unit cell volume of TlCdCl_3_ crystals is slightly larger than that of TlCdCl_3_:Bi crystals, which was not observed for the TlCdI_3_ and TlCdI_3_:Bi. Therefore, due to the r_Bi_ > r_Tl_, the more complex character of defect formation in the TlCdCl_3_:Bi crystal is possible: (Tl,Bi)_0.998(1)_Bi_(i)0.005(1)_CdCl_3_; 0 → Bi_Tl_^×^ + V_Tl_^n^′ + Bi_i_^n•^ (Equation (26)). It should be noted that here we deal with the main point defects that can be detected by XRD with the corresponding sensitivity of the experiment. The greater content of interstitial Bi atoms near Tl atoms in the TlCdI_3_:Bi^1+^ structure compared to the TlCdCl_3_:Bi^1+^ is due to the larger size of I^1-^ ions (*R*_I_^VI^ = 2.20 Å [84]) compared to Cl^1−^ ions (*R*_Cl_^VI^ = 1.81 Å [84]), i.e., a peculiar implementation of the “intermediary rule” is observed [7]. Similar behavior was revealed for the CsCdCl_3_ and CsCdBr_3_ crystals.

**Figure 17:**
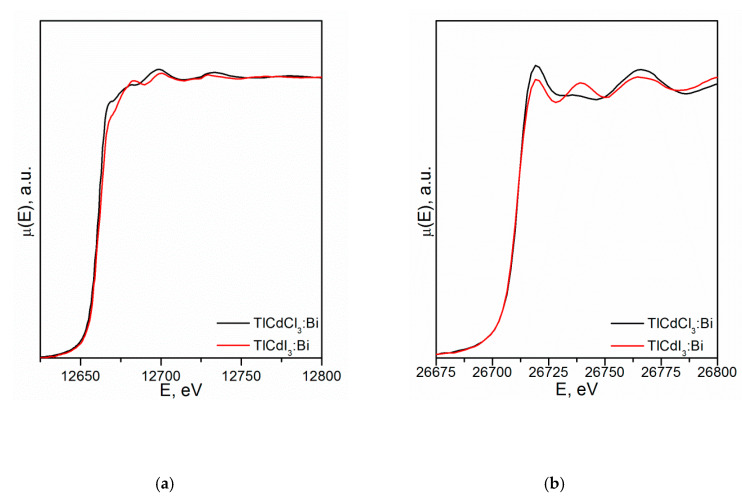
XANES spectra measured (**a**) at the Tl L_3_-edge and (**b**) at the Cd K-edge for TlCdCl_3_:Bi and TlCdI_3_:Bi crystals.

The XANES spectra measured at the Tl L_3_-edge for the TlCdCl_3_:Bi and TlCdI_3_:Bi samples have different intensities of the “white line” and some shifts of curves along the energy scale, but the spectra of both samples are similar in general (Figure 17).

Such differences may be due to the fact that, for similar local structures, in one of the samples, Tl^1+^ ions are coordinated by Cl^1−^ ions, and in the other, by I^1−^ ions.

**Figure 18:**
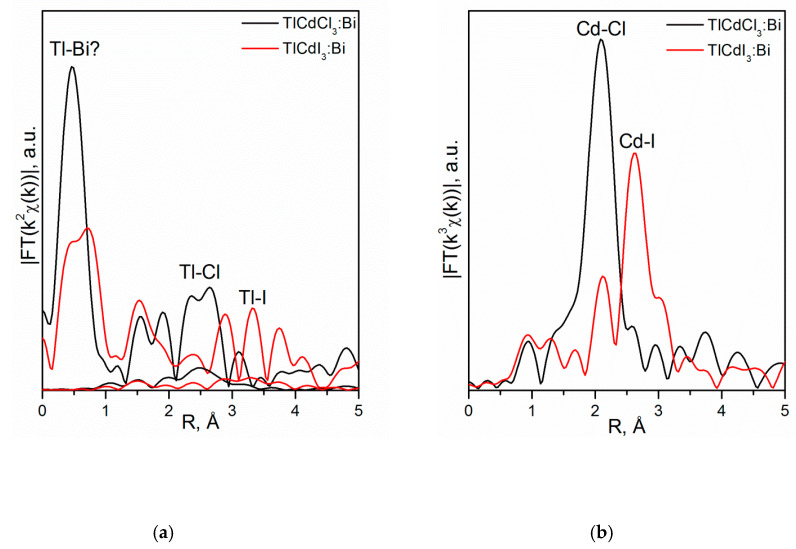
EXAFS Fourier transform curves measured (**a**) at the Tl L_3_-edge and (**b**) at the Cd K-edge for TlCdCl_3_:Bi and TlCdI_3_:Bi crystals.

The EXAFS Fourier transform curves measured at the Tl L_3_-edge for the TlCdX_3_:Bi^1+^ samples contain a series of peaks corresponding to the environment of Tl^1+^ ions by the *X*^1−^ ions (Figure 18a). Table 11 contains the results of EXAFS fitting the Tl environment using three nonequivalent paths of photoelectron scattering on Cl or I atoms.

The fitting results in the significantly reduced (*d*_EXAFS_ = 2.53 Å and 2.73 Å) and increased (*d*_EXAFS_ = 3.86 Å and 4.02 Å) several interatomic distances compared with the XRD data for the TlCdCl_3_:Bi^1+^ and TlCdI_3_:Bi^1+^ samples, respectively (Table 11). This may indicate the presence of vacancies either at the Cl or Tl site, the latter being consistent with the XRD data. A decrease in the Fourier filtering to *R*_bkg_ ~0.2 Å allows revealing an intense peak at very small interatomic distances (~0.6 Å) (Figure 18), which may indicate the presence of Bi atoms at an ultrashort distance (1 Å or less) from Tl (Bi_i_^n•^).

The EXAFS Fourier transforms measured at the Cd K-edge for the TlCd*X*_3_:Bi samples have one significant peak corresponding to the chlorine or iodine environment of Cd atom. There are four nonequivalent Cd–Cl distances in the TlCdCl_3_ structure; however, three of them lie in the range 2.6–2.7 Å. Since an error in determining the non-equivalent, but similar distances can exceed 0.05 Å, two independent scattering paths are reasonably to be considered. As a result, the coordination numbers are close to the volume value CN Cl = 6, and interatomic distances correspond (with the accuracy with which they can be estimated) to the XRD data.

Different behavior of Bi ions in the TlCdCl_3_:Bi and TlCdI_3_:Bi structures (Bi^1+^ ions occupy interstitial site (Bi_i_^•^) or Tl site (Bi_Tl_^×^)) and in the CsCdCl_3_:Bi and CsCdBr_3_:Bi structures (Bi^3+^ ions replace Cd^2+^ ions (Bi_Cd_^•^)) is due to the chemical similarity of Tl^1+^ (r_Tl_^XII^ = 1.70 Å; χ_Tl_ = 1.5) and Bi^1+^ (r_Bi_^XII^ = 1.903 Å; χ_Bi_^1+^ = 1.4) ions, in contrast to Cs^1+^ and Bi^1+^ ions.

Thus, the XRD and XAS study of CsCdBr_3_:Bi and CsCdCl_3_:Bi, TlCdCl_3_:Bi and TlCdI_3_:Bi crystals and crystal–chemical analysis of the results obtained allowed to establish actual crystal compositions. The expected presence of Bi^1+^ ions at the Cs^1+^ and Tl^1+^ sites in the Bi-doped CsCdBr_3_ and CsCdCl_3_, TlCdCl_3_ and TlCdI_3_ structures was not confirmed [111,112], which is explained by the theory of isomorphism.

Photoluminescence spectra of TlCdCl_3_:Bi and TlCdI_3_:Bi single-crystal samples excited with light with different wavelengths are given in Ref [112]. The photoluminescence bands were correlated with point defects determined by the XRD analysis and XAS. In contrast to photoluminescence spectra of CsCdCl_3_:Bi [101] and CsCdBr_3_:Bi [111], containing a single band with λ ~1000 nm, two bands with λ ~1025 and ~1253 nm and one band with λ = 1175 nm were detected for the TlCdCl_3_:Bi and TlCdI_3_:Bi, respectively. The bands with λ ~1000 nm correspond to the Bi^1+^ cation (Bi_Tl_)^×^, the band with λ ~1253 nm is associated with the interstitial Bi^1+^ cations (Bi_i_^n^^•^) and the wide photoluminescence band with λ ~1175 nm is probably due to a high concentration of Bi^1+^ interstitials (Bi_i_^n^^•^) in the TlCdI_3_:Bi.

The characteristic lifetimes are 150 µs and 170 µs for the TlCdCl_3_:Bi. It is assumed that the appearance of IR photoluminescence bands in the TlCdCl_3_:Bi is apparently due to the optical transitions between the energy levels of the Bi^+^ cation located in a crystalline environment (field), similar to the KMgCl_3_:Bi crystals, which also possess long-lived luminescence [89].

## 5. Summary

Actual compositions, structural features and functional properties of complex oxides with the scheelite and eulytin structures as well as halides with the perovskite structure and other compounds with similar complex compositions were studied, analyzed and explained. The objects of the investigation were examined with research facilities (neutron diffraction, synchrotron X-ray powder diffraction, single-crystal X-ray diffraction), structurally characterized (statistical structure: analysis of cell parameters, atomic displacements, residual electron (nuclear) density, site occupancies), studied using X-ray absorption spectroscopy (local structure: coordination environment of matrix ions and activator ions and their formal charges) and analyzed using concepts of fundamental crystal chemistry. Additional consideration of other single-crystal objects, which were studied by the developed methodology, made it possible to clarify, confirm, expand and generalize the results obtained and reveal structural effects caused by various factors and, in many cases, due to the local structure:

•**Compositional factor (volume):** Different formal charges, structural localization and concentration of activator ions depending on the composition of the matrices.

*Example*: The Bi dopant ions were introduced in the form of a mixture (Bi + Bi*X*_3_) into the matrices with the initial compositions CsCd*X*_3_ (*X* = Cl, Br) and TlCd*X*_3_ (*X* = Cl, I):

- CsCd*X*_3_ (*X* = Cl, Br) (perovskite family, space group *P*6_3_/*mmc*) with the refined actual compositions (Cs(1)_0.989(1)_⎕_0.011_)Cs(2)(Cd^2+^(1)_0.995(2)_Bi^3+^(1)_0.005_)(Cd^2+^(2)_0.996(2)_Bi^3+^(2)_0.004_)Cl_6_ (*a* = 7.418(4), *c* = 18.39(3) Å) and (Cs_0.976(10)_⎕_0.024_)(Cd_0.967(47)_Bi^3+^_0.033_)Br_3_ (*a* = 7.675(3), *c* = 6.722(3) Å) (⎕-vacancies);
- TlCd*X*_3_ (*X* = Cl, I) (space group *Pnma*) with the refined actual compositions Tl^1+^_0.980(12)_Bi^1+^_(i)0.014(12)_CdI_3_ and (Tl^1+^,Bi^1+^)_0.998(1)_Bi^1+^_(i)0.005(1)_CdCl_3_.

The refined crystal compositions with the arrangment of the matrix ions, vacancies, the Bi^1+^ and/or Bi^3+^ activator ions over the crystallographic sites made it possible to explain different spectral-luminescent behavior [91,101,102,111,112] and an occurrence of two (TlCdCl_3_:Bi^1+^) and one (TlCdI_3_:Bi^1+^) optical centers for Bi^1+^ ions [112] and one for Bi^3+^ ions (CsCd*X*_3_:Bi^3+^) [111].

•**Compositional factor (surface):** Different processes of defect formation depending on the compositions of the crystal surface and nanoparticles containing activator ions.

*Example*: Titanium (IV) oxide TiO_2_ nanoparticles with anatase structure or η-modification TiO_2−x_ × *n*H_2_O were introduced into the solution to grow KH_2_PO_4_:Ti^4+^ crystals (space group $I\bar{4}2d$) by the hydrothermal method. Depending on the prehistory of TiO_2_ (chloride or sulfate synthesis method; anatase structure or η-modification) and the symmetry of surfaces of growth sectors (prismatic (*Pr*) or pyramidal (*P*); the system of free bonds is greater on the surface of the *Pr* sector than on the *P* one) in the KH_2_PO_4_:Ti^4+^ single crystals, different types of point defects were found (⎕-vacancies):

- (K^1+^_0.994(18)_⎕_0.006_)Ti^4+^_(i)0.006_(H_0.964(22)_⎕_0.036_)_2_[(P^5+^O_4_)_0.946_(S^6+^O_4_)_0.054_] (sulfate synthesis of η-TiO_2_; *P*),
- (K^1+^_0.963(10)_⎕_0.037_)Ti^4+^_(i)0.037_(H_0.914(12)_⎕_0.086_)_2_[(P^5+^O_4_)_0.939_(S^6+^O_4_)_0.061_] (sulfate synthesis of η-TiO_2_; *Pr*), described by a quasi-chemical reaction: 0 ^→^ V_K_′ + Ti^n^^•^ + S_P_^•^ + V_H_′ (Equation (27));
- (K^1+^_0.994(2)_⎕_0.004_)Ti^4+^_(i)0.002_H_2_(P^5+^O_4_) (chloride synthesis of anatase*; P*),
- (K^1+^_0.990(6)_⎕_0.010_)Ti^4+^_(i)0.003_H_2_(P^5+^O_4_) (chloride synthesis of anatase*; Pr*), described by a quasi-chemical reaction: 0 ^®^ V_K_′ + Ti^n^^•^ (Equation (28)).

The shift in the electron density in the O–H···O hydrogen bond towards the short O–H bond, caused by vacancies in the H site, the content of which depends on the (P^5+^O_4_):(S^6+^O_4_) ratio, decreases the dielectric constant of crystals (greater for the η-TiO_2_) obtained by the sulfate method [168].

•**Size factor**: Different structural localization of activator ions depending on the correlation between sizes of matrix and activator ions.

*Example*: The SrMoO_4_ crystals (scheelite family, space group *I*4_1_/*a*) doped with the over stoichiometric amount of *RE^3+^* ions (*RE* = Ho, Tm) in the form of *RE*NbO_4_ showed different actual compositions due to different sizes (r, Å) of Ho^3+^ and Sr^2+^, Tm^3+^ and Sr^2+^ ions (⎕-vacancies):

- (Sr^2+^_0.998(2)_Ho^3+^_0.002_)[(Mo^6+^,Nb^5+^)_0.998_⎕_0.002_]Nb^5+^_(i)0.002_(O_3.96(3)_)(1)(O_0.002+*x*_)(2) (Δr/r_min_ < 2 5%),
- (Sr^2+^_0.992(3)_⎕_0.008_)Tm^3+^_(i)0.008_[(Mo^6+^,Nb^5+^)_0.992_⎕_0.008_]Nb^5+^_(i)0.008_(O_3.80(4)_)(1)(O_0.008+*x*_)(2) (Δr/r_min_ > 25%) with new sites for interstitial oxygen–O(2). The greater disordering of the SrMoO_4_:Tm structure made it possible to propose this crystal as a promising material for tunable (~2 μm) lasers pumped by a diode laser (1700 nm) [95].

•**Concentration factor:** Different coordination polyhedra or different formal charges of activator ions depending on their content in the crystals.

*Example* *1:* Activation of Bi_4_Ge_3_O_12_ (BGO) crystals (eulytin structure, space group $I\bar{4}3d$) with the over stoichiometric amount of Dy^3+^ ions in the form of Dy_2_O_3_ reduces the symmetry of the local regions (~13%) of the crystal with a change in the local environment of cations:

- (Bi^3+^_3.993(5)_Dy_0.007_)Ge_3_O_12_ (BGO:0.1% Dy; the DyO_7_ polyhedron is a derivative of a trigonal prism; the BiO_5_ polyhedron is a defective octahedron),
- (Bi^3+^_3.972(5)_Dy_0.028_)Ge_3_O_12_ (BGO:1.0% Dy^3+^; the DyO_5_ polyhedron is a semi-octahedron or polyhedron derived from a trigonal prism; the BiO_6_ polyhedron is a distorted octahedron) [120].

*Example* *2:* An increase in the Ni^3+^ content (activator ions were introduced in the form of Ni_2_O_3_ over stoichiometry) in the composition of (Sr_0.61_Ba_0.39_)Nb_2_O_6_ (SBN) crystals with congruent melting (a family of tetragonal bronzes, space group *P4bm*) contributes to the transition Ni^3+^→Ni^2+^ (⎕-vacancies):

- SBN:0.5% Ni—Sr(1)_0.284(1)_Sr(2)_0.341(2)_Ba(2)_0.362(2)_Nb(1)(Nb(2)_0.975(1)_⎕_0.025_)Ni^3+^_(i)0.025_(O_5.962_⎕_0.038_);
- SBN:1.0% Ni- Sr(1)_0.300(1)_Sr(2)_0.384(1)_Ba(2)_0.347(2)_Nb(1)(Nb(2)_0.972(1)_⎕_0.028_)Ni^2+^_(i)0.028_(O_5.982(8)_⎕_0.018_).

The degree of distortion of the Nb(1)O_6_ octahedron decreases with increasing Ni content and correlates with second-order nonlinear susceptibility. In the (Sr,Ba)Nb_2_O_6_:Ni crystals, additional energy levels of impurity ions increase the photo-refractive properties and shift the edge of the absorption band to the long-wavelength region [117].

*Example* *3:* Investigation of the scheelite-type (Bi_2-*x*_Ce*_x_*)(MoO_4_)_3_ crystals [169] in the concentration ranges 0 ≤ *x* ≤ 0.25 and 1.0 ≤ *x* ≤ 1.5 by X-ray absorption spectroscopy at the Bi and Ce L_3_-edges and Mo K-edge showed that an increase in *x* results in Ce coordination number increase from 8 (for *x* ≤ 0.25) to 10 (for *x* ≥ 1.0). At the same time, the Bi coordination number decreases from 8 to 6 (4 + 2).

•**Coordination factor (matrix-“host”):** The prevailing role of coordination polyhedra of the structure (crystalline matrix) in the realization and/or localization of ions with different formal charges.

*Example* *1:* The refined composition of the Bi^3+^_4_Ge_3_O_12_ (BGO) crystal was found to be (Bi^3+^_3.994(40)_⎕_0.006_)(Ge_2.980(48)_Bi^5+^_0.020_)O_12_ (⎕-vacancies) with the distribution of Bi^3+^ and Bi^5+^ ions, respectively, over the distorted octahedral and tetrahedral sites of the eulytin structure (space group $I\bar{4}3d$), which is due to the impossibility of Bi^3+^ ions, due to their large size (r ~0.89 Å), to form the tetrahedra, even taking into account cationic vacancies in these sites [110]. A similar situation is observed for sillenite family crystals (space group *I*23) with the general composition Bi_24_(Bi,Mn^4+^)_2_O_40−δ_ [151].

*Example* *2:* The scheelite family crystals (space group *I*4_1_/*a*) with the initial composition (Na_0.5_La_0.5–2x_Ce^4+^_0.15_Er_0.15_)MoO_4_ (i.e., crystals are co-doped with the Ce^4+^ and Er^3+^ ions, however, their composition is closer to solid solutions) have actual composition Na_0.5_La_0.345_Ce^3+^_0.15_Er_0.005_)MoO_4_. Moreover, Ce^4+^O_2_ from the initial charge was detected on the dislocations of the unannealed crystal, while CaF_2_-type CeO_2_ crystals (space group *Fm*3*m*) with well-formed faces and structural-geometric correspondence with scheelite structure were formed on the inner surfaces of the annealed crystal [107].

•**Coordination factor (activator ion—“guest”):** Formation of intrinsic coordination polyhedra of activator ions with or without a change in crystal symmetry.

*Example* *1:* Local symmetry reduction from monoclinic (space group *C*2/*c*) to triclinic (space group *P*1 or $P\bar{1}$) was observed by X-ray diffraction analysis for the huntite-family La^3+^Sc^3+^_3_(BO_3_)^3+^_4_ crystals both nominally pure and doped with the Cr^3+^ ions [170]. In the latter case, about 15% of the reflections with *I* < 3σ(*I*) are not indexed in the monoclinic symmetry [170], which is associated with the Cr^3+^ activator ions. These ions form the regular CrO_6_ octahedra in contrast to the distorted ScO_6_ octahedra in the huntite matrix due to the electronic structure of Cr^3+^ ions (non-binding d_ε_^3^ configuration is symmetric with respect to the octahedral ligand field). This should result in a local change in the symmetry of the (Sc/Cr^3+^)O_6_ polyhedra and a possible change in the symmetry of the whole crystal [155,171].

*Example* *2:* Structure of Ca_3_(VO_4_)_2_ crystals (CVO) (whitlockite family; space group *R*3*c*) contains five crystallographic sites for Ca^2+^ ions, one of which is split (Ca5 and Ca5*A*): Ca1 and Ca3 are bicapped trigonal prisms, Ca2 is a monocapped trigonal prism, Ca4 is an octahedron, Ca5 + Ca5*A* is a defective highly distorted octahedron. In the Ca^2+^_3_(VO_4_)_2_:Tm^3+^ structure, the Tm^3+^ activator ions retain their structural individuality with the CN Tm = 6 (octahedron): the Tm^3+^ ions partially enter the Ca4 octahedral site and form their octahedral environment at the Ca3 site with an additional oxygen atom. Two pronounced peaks (λ = 470.5 and 473.5 nm) on the absorption spectra (77 K), two different luminescence spectra for different excitations, two components with different lifetimes (1380 μs and 370 μs) in the luminescence decay kinetics were revealed for the CVO:1.0% Tm crystals. All this indicates the presence of two different optical centers of Tm^3+^ ions in the Ca_3_(VO_4_)_2_ crystal with excellent spectroscopic properties, which is completely consistent with the structural data [172,173,174]. For doped Ca_3_(VO_4_)_2_:Tm^3+^ crystals and solid solutions with the general composition (Ca^2+^,*RE*^3+^)_3_(VO_4_)_2_ [175,176,177], different structural behavior of *RE*^3+^ ions is observed: depending on the type of *RE*^3+^ ions, different distribution over crystallographic sites is revealed in solid solutions (Tm^3+^ enter only the octahedral sites in CVO:Tm).

•**Crystallochemical** **factor:** The disorder-order transition with a reduction of the crystal symmetry:

*Example* *1:* For scheelite family crystals with the charge compositions the following symmetry reduction is observed:

- (Na,Gd)WO_4_:Yb—from the space group *I*4_1_*/a* to $I\bar{4}$ in the local region (up to 70%) of the crystal with increasing Yb content (up to 10%);
- (Na_0.5_Gd_0.5_)MoO_4_:10% Yb—from the space group *I*4_1_*/a* to $P\bar{4}$ [109];
- (Na_0.5_Gd_0.5_)WO_4_:0.035% Tm—from the space group *I*4_1_*/a* to *P*4_2_*/nnm* [17];
- (Na_0.5_Gd_0.5_)WO_4_:0.05% Tm—from the space group *I*4_1_*/a* to $I\bar{1}$ [17].

An X-ray diffraction study of crystals with the nominal compositions (Na_0.5_Gd_0.5_)MoO_4_:3% Yb and (Na_0.5_Gd_0.5_)MoO_4_:10% Yb showed a multiple increase in the unit cell parameters [109]. A formation of a partially ordered noncentrosymmetric phase in the area of stability of the disordered centrosymmetric phase was observed for scheelite family solid solutions: symmetry reduction from the space group *I*4_1_*/a* (*x* = 0.2 ‒ 1.0) to $I\bar{4}$ (*x* = 0) was found in the local region (20%) of PbMo*_x_*W_1-*x*_O_4_ crystals due to the ordering of oxygen vacancies [105].

*Example* *2:* The ordering of cations over crystallographic sites in the garnet family crystals with the general composition {Yb_,_Sc}_3_ [Sc,Yb] _2_Ga_3_O_12_ lowers the symmetry from the space group *Ia*3*d* to *P*4_2_32 (most likely) in the local region (46%) of the crystal [8].

*Example* *3:* Analysis of diffraction reflections of sillenite family solid solutions (space group *I*23) with the refined compositions Bi_24_(Si^4+^_0.04(8)_Bi^3+^_0.60_Mn^4+^_1.36_)(O_39.70_⎕_0.30_), Bi_24_(Si^4+^_0.9(1)_Mn^4+^_1.1_)O_40_, Bi_24_(Si^4+^_0.17(1)_Bi^3+^_0.01_Mn^4+^_1.82_)(O_39.93(5)_⎕_0.07_) (⎕-vacancies) allowed to reveal additional reflections *hkl* with *h + k + l ≠ 2n,* 0*kl* with *k + l ≠ 2n, hhl* with *l ≠ 2n, h*00 with *h ≠ 2n*, which can be indexed in the space group *P*23. As a result, the actual composition of the sample Bi_24_(Si^4+^_0.17(1)_Bi^3+^_0.01_Mn^4+^_1.82_)(O_39.93(5)_⎕_0.07_) (space group *I*23) can be written as Bi_24_(Bi_0.04(2)_Si_0.05_Mn_0.91_)(Bi_0.08(2)_Si_0.30_Mn_0.62_)(O_39.93(5)_⎕_0.07_) (space group *P*23) (⎕-vacancies), i.e., a symmetry transition leads to split crystallographic sites of atoms [178].

Naturally, the abovementioned structural effects are roughly attributed to different factors. For example, Example 3, related to the Concentration factor, may also be attributed to Coordination factor (activator ion—“guest”).

The above examples indicate that the structural effects observed in fundamentally different crystal systems are of a general nature and may appear in other systems as a result of the formation of solid solutions and doping of crystals. To reveal the structural effects and features of the crystalline sample, the use of diffraction methods in combination with X-ray absorption spectroscopy, followed by a crystal–chemical analysis of the results, is required.

## 6. Conclusions

The specific structural features of multifunctional single-crystal materials of scheelite, perovskite, eulytin and other families (huntite, sillenite, whitlockite, garnet, tetragonal bronzes) grown by the melt methods are described. The combined application of X-ray diffraction and X-ray absorption spectroscopy, followed by a crystal–chemical analysis of the results obtained, is necessary to reveal different structural effects in pure and doped single crystals of a wide range of compositions and structures. The reasons for the appearance of specific structural effects and phenomena are discussed.

The systematized results of the investigation of pure and doped functional single crystals of different families allow establishing the correlations between growth conditions, composition, structure and functional properties. As a result, the operational parameters of crystals are reasonably associated with their fundamental characteristics (composition, local and statistical structure, etc.) depending on the history of the samples (the composition of the initial charge, method of doping, growth and post-growth conditions, etc.). This allows revealing the most successful compositions and combinations of crystalline matrix and active ions for further directed growth of the material with the required characteristics.

## 7. Patents

1. Kuzmicheva, G.M.; Podbelsky, V.V.; Chuykin, N.K.; Kaurova, I.A. A software package for studying the dynamics of changes in the structural parameters for compounds with different symmetries. Certificate of state registration of software package No. 2017619941 [Russ.].
2. Kuzmicheva, G.M.; Podbelsky, V.V.; Nemashkalo, M.A. A software package for calculating the dynamics of changes in coordination polyhedra during isomorphic substitution in simple scheelites. Certificate of state registration of software package No. 2017610700 [Russ.].

## Figures and Tables

**Table 1:** Symmetry and refined compositions of PbMoO_4_ (PMO) and PbWO_4_ (PWO) crystals according to single-crystal X-ray (XRD) and neutron diffraction (ND) analysis.

Sample	Designation	Growth Atmosphere	Color	Space Group	Refined Composition ^1^
PbMoO_4_	PMO-A	Air	Yellow	*I*4_1_/*a*	(Pb_0.976(1)_⎕_0.024_)MoO_4.00(3)_
PbWO_4_	PWO-A			$I\bar{4}$	PbW(O_3.982(3)_⎕_0.018_) PbW(O_3.94(4)_⎕_0.06_) ^2^
PbMoO_4_	PMO-N_2_	Nitrogen		*I*4_1_/*a*	(Pb_0.960(1)_⎕_0.040_)MoO_4.00(4)_
PbWO_4_	PWO-N_2_			$I\bar{4}$	Pb(W_0.996(1)_⎕_0.004_)(O_3.855(3)_⎕_0.145_) Pb_0.999(1)_W(O_3.93(4)_⎕_0.07_) ^2^

^1^ Vacancies are marked with a square (⎕). ^2^ Neutron diffraction (ND) data.

**Table 2:** Refined compositions of PbMo_x_W_1−x_O_4_ (PMWO) crystals according to single-crystal X-ray (XRD) and neutron diffraction (ND) analysis.

Sample	Designation	Color	Unit Cell Parameters (*a, c,* Å) and Cell Volume (*V*, Å^3^)	Refined Composition ^1^
PbMo_0.2_W_0.8_O_4_	PMWO-1	Colorless	5.4411(4) 12.0261(12) 356.04 5.4468(2) ^2^ 12.0679(4) ^2^ 358.03 ^2^	(Pb^2+^_0.980(5)_Y^3+^_0.020_)(Mo^6+^_0.300(5)_W^6+^_0.700_)(O_3.990(8)_⎕_0.010_) or (Pb^2+^_0.980_Y_0.020_)[(Mo^6+^,W^6+^)_0.960_Nb^5+^_0.040_](O_3.990(8)_⎕_0.010_) (Pb^2+^_0.972_Y^3+^_0.028(4)_)(Mo^6+^_0.490_W_0.482(8)_Nb^5+^_0.028_)O_4_^2^
PbMo_0.5_W_0.5_O_4_	PMWO-2		5.4350(5) 12.0926(15) 357.21 5.4406(3) ^2^ 12.0700(3) ^2^ 357.27 ^2^	(Pb^2+^_0.975(8)_Y^3+^_0.025_)(Mo^6+^_0.536(5)_W^6+^_0.464_)(O_3.985(10)_⎕_0.015_) or (Pb^2+^_0.975_Y_0.025_)[(Mo^6+^,W^6+^)_0.945_ Nb^5+^_0.055_](O_3.985(10)_⎕_0.015_) (Pb^2+^_0.960_Y^3+^_0.040(4)_)(Mo^6+^_0.519_W_0.441(8)_Nb^5+^_0.040_)O_4_^2^
PbMo_0.8_W_0.2_O_4_	PMWO-3		5.4398(3) 12.0534(9) 356.68 5.4296(3) ^2^ 12.0629(4) ^2^ 355.62 ^2^	(Pb^2+^_0.930(10)_Y^3+^_0.070_)(Mo^6+^_0.770(8)_W_0.230_)(O_3.960(20)_ ⎕_0.040_) or (Pb^2+^_0.930(10)_Y^3+^_0.070_)[(Mo^6+^,W^6+^)_0.850_Nb_0.150_](O_3.960(20)_ ⎕_0.060_) (Pb^2+^_0.950_Y^3+^_0.050(8)_)[(Mo^6+^_0.719_W_0.183(8)_Nb^5+^_0.100_](O_3.980(10)_⎕_0.020_) ^2^

^1^ Vacancies are marked with a square (⎕). ^2^ Neutron diffraction (ND) data.

**Table 3:** Color and refined compositions of PbMoO_4_ and PbMoO_4_:Nd^3+^ crystals according to single-crystal X-ray (XRD) and neutron diffraction (ND) analysis.

Number	Sample	Color	Refined Composition ^1^
1	PbMoO_4_	Yellowish transparent	Pb^2+^(Mo_0.992(4)_⎕_0.008_)O_4_ Pb^2+^ Mo_1.000(16)_O_3.960(44)_^2^
2	PbMoO_4_:Nd_2_O_3_	Lilac yellow with predominant yellow	(Pb^2+^_0.970(4)_Nd^3+^_0.030_)Mo^6+^O_4_ (Pb^2+^_0.865(45)_Nd^3+^_0.085_⎕_0.050_)Mo^6+^O_4_^2^
3	PbMoO_4_:NdNbO_4_	Lilac	(Pb^2+^_0.980(6)_Nd^3+^_0.020_)(Mo^6+^_0.980_Nb^5+^_0.020_)O_4_ (Pb^2+^_0.968(16)_Nd^3+^_0.032_)(Mo^6+^_0.970(20)_Nb^5+^_0.030_)O_4_^2^
4	PbMoO_4_:Nd_2_(MoO_4_)_3_	Lilac yellow with predominant yellow	(Pb^2+^_0.980(4)_Nd^3+^_0.020_)Mo^6+^O_4_ (Pb^2+^_0.964(15)_Nd^3+^_0.025_⎕_0.011_)Mo^6+^O_4_^2^
5	PbMoO_4_:(Na_0.5_Nd_0.5_)MoO_4_	Deep lilac	(Pb^2+^_0.960(6)_Nd^3+^_0.020(8)_Na^+1^_0.015_)Mo^6+^O_4_ (Pb^2+^_0.935(12)_Nd^3+^_0.033_Na^+1^_0.033_)Mo^6+^O_4_^2^

^1^ Vacancies are marked with a square (⎕). ^2^ Neutron diffraction (ND) data.

**Table 4:** Refined compositions of SrMoO_4_, SrMoO_4_:Ho, SrMoO_4_:Tm crystals according to single-crystal X-ray diffraction (XRD) and X-ray absorption spectroscopy (EXAFS/XANES).

Sample	Designation	Refined Composition (XRD)	Refined Composition ^1^ (XRD + EXAFS/XANES)
SrMoO_4_	SMO	SrMoO_4_	-
SrMoO_4_:HoNbO_4_ (0.1 wt%)	SMO:0.1Ho	(Sr_0.996(4)_Ho_0.004_)MoO_4_	-
SrMoO_4_:HoNbO_4_ (0.5 wt%)	SMO:0.5Ho	(Sr_0.992(3)_Ho_0.008_)MoO_4_	-
SrMoO_4_:HoNbO_4_ (1.0 wt%)	SMO:1.0Ho	(Sr_0.998(2)_Ho_0.002_) [(Mo_0.998(2)_⎕_0.002_)(Nb_0.002_)_i_]O_3.96(3)_	(Sr^2+^_0.998(2)_Ho^3+^_0.002_) [(Mo^6+^,Nb^5+^)_0.998_⎕_0.002_][(Nb^5+^_0.002_)_i_] [(O_3.96(3)_)(1)(O_0.002+x_)(2)]
SrMoO_4_:TmNbO_4_ (0.5 wt%)	SMO:0.5Tm	[Sr_0.996(2)_⎕_0.004_)(Tm_0.004_)_i_] [Mo_0.996(3)_⎕_0.004_)(Nb_0.004_)_i_]O_4_	-
SrMoO_4_:TmNbO_4_ (1.0 wt%)	SMO:1.0Tm	[(Sr_0.992(3)_⎕_0.008_)(Tm_0.008)i_)] [(Mo_0.992(3)_⎕_0.002_)(Nb_0.008)i_)]O_3.80(4)_	[(Sr^2+^_0.992(3)_⎕_0.008_)(Tm^3+^_0.008i_)] [(Mo^6+^,Nb^5+^)_0.992_⎕_0.008_][(Nb^5+^_0.008_)_i_] [(O_3.80(4)_)(1)(O_0.008+x_)(2)]

^1^ Vacancies are marked with a square (⎕).

**Table 5:** The results of one-sphere EXAFS fit for strontium molybdate SrMoO_4_ (SMO), SMO:Ho and SMO:Tm: coordination numbers (CN Mo) and Mo–O distances (*d*_EXAFS_, Å).

Sample	CN Mo	*d*_EXAFS_, Å
SMO	4	1.768(5)
SMO:0.1Ho	4.2(7)	1.792(5)
SMO:0.5Ho	4.1(7)	1.794(6)
SMO:1.0Ho	3.8(3)	1.799(4)
SMO:0.5Tm	4.1(7)	1.791(7)
SMO:1.0Tm	3.9(3)	1.787(5)

**Table 6:** Growth and post-growth conditions, color and refined compositions of (Na, Gd)MoO_4_ (NGM) crystals according to single-crystal X-ray diffraction (XRD) analysis.

Sample	Designation	Color	Growth/Annealing Atmosphere	Refined Composition ^1^
(Na_0.5_Gd_0.5_)MoO_4_	NGM-I ^2^	Dark gray	N_2_ + 2 vol% O_2_/–	(Na_0.493(3)_Gd_0.507_)Mo(O_3.920(5)_⎕_0.080_)
(Na_0.5_Gd_0.5_)MoO_4_	NGM-A ^2^	Slightly yellow	N_2_ + 2 vol% O_2_/Air	(Na_0.495(3)_Gd_0.505_)Mo(O_3.996(8)_⎕_0.004_)
(Na_0.5_Gd_0.5_)MoO_4_	NGM-Ar(1) ^3^ NGM-Ar(2) ^2^ (NGM 1:1)	Dark gray Almost colorless	Ar/–	(Na_0.489(4)_Gd_0.511_)(Mo_0.995(3)_⎕_0.005_)(O_3.915(10)_⎕_0.085_) (Na_0.498(2)_Gd_0.502_)(Mo_0.999(4)_⎕_0.001_)O_4_
(Na_2/7_Gd_4/7_)MoO_4_	NGM-2/7 ^2^ (NGM 1:2)	Yellow	Air/–	(Na_0.348(8)_Gd_0.528_⎕_0.124_)(Mo^6+^_0.996(3)_⎕_0.004_)O_4_
(Na_6/15_Gd_8/15_)MoO_4_	NGM-6/15 ^3^	Dark gray	Air/–	(Na_0.300(8)_Gd_0.576_⎕_0.124_)Mo(O_3.880(10)_⎕_0.120_)

^1^ Vacancies are marked with a square (⎕). ^2^ Mo*K*_α_-radiation. ^3^ Ag*K*_α_-radiation.

**Table 7:** The results of two-sphere EXAFS fit for NGM 1:1 and NGM 1:2 crystals (*k*-range: 2–13 Å^−1^): coordination number, CN; interatomic distance between the central atom and the next neighbors (the length for single scattering path), *d*, Å; the Debye–Waller factor, σ^2^. The braces are combined atoms belonging to the same coordination sphere.

CaMoO4 [14]		Nominal Compositions and their Designation							
		(Na_1/2_Gd_1/2_)MoO_4_ (NGM 1:1)		(Na_2/7_Gd_4/7_⎕_1/7_)MoO_4_ (NGM 1:2)					
CN	*d*, Å	CN for Scattering Paths	*d*, Å/σ^2^, Å^2^	CN for Scattering Paths			*d*, Å/σ^2^, Å^2^		
		The parameters of local structure obtained from Gd L_3_-edge EXAFS modeling							
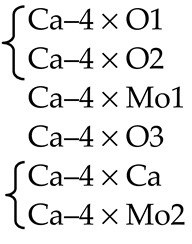	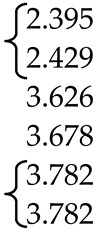	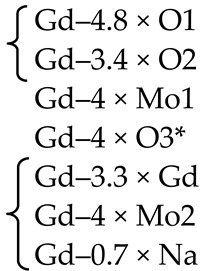	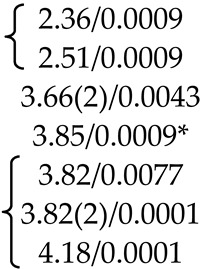		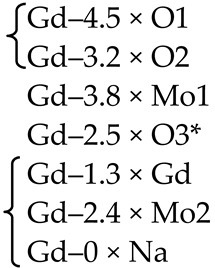			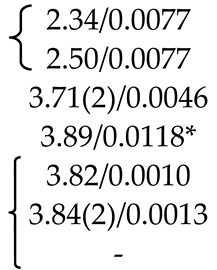	
		The parameters of local structure obtained from Mo K-edge EXAFS modeling							
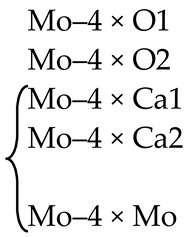	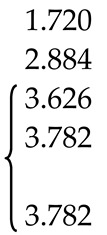	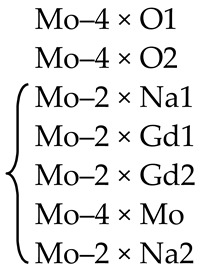	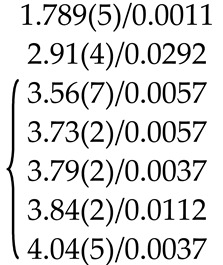			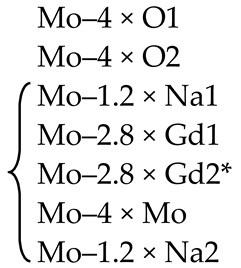			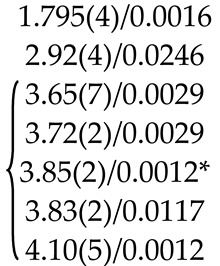

**Table 8:** Refined compositions of the BGO and BGO:Dy crystals according to single-crystal X-ray diffraction (XRD) analysis.

Initial Charge Composition	Designation	Color	Refined Composition ^1^
Bi_4_Ge_3_O_12_	BGO(C)	colorless	(Bi^3+^_3.994(40)_⎕_0.006_)[Ge_2.980(30)_Bi^5+^_0.020_)O_12.00_
Bi_4_Ge_3_O_12_	BGO(P)	pink	(Bi^3+^_3.987(45)_⎕_0.013_)(Ge_2.988(35)_Bi^5+^_0.012_)(O_11.98(5)_⎕_0.02_)
Bi_4_Ge_3_O_12_:1.0% Dy	BGO:1.0Dy	colorless	(Bi^3+^_3.952(47)_Dy_0.048_)Ge_3_O_12_
Bi_4_Ge_3_O_12_:0.1% Dy	BGO:0.1Dy	colorless	(Bi^3+^_3.996(11)_Dy_0.004_)Ge_3_O_12_

^1^ Vacancies are marked with a square (⎕).

**Table 9:** Results of single-sphere EXAFS fit for BGO:Dy crystals.

Initial Charge Composition	Coordination Number (CN)		Interatomic Distance, Å
Bi_4_Ge_3_O_12_:0.1Dy	Dy-O	7.6	2.21
	Bi-3O	2.5	2.13
	Bi-3O	2.5	2.58
Bi_4_Ge_3_O_12_:1.0Dy	Dy-O	4.7	2.31
	Bi-3O	2.9	2.13
	Bi-3O	2.9	2.57

**Table 10:** Results of EXAFS fit at the Cd K-edge for the CsCdBr_3_, CsCdBr_3_:Bi and CsCdCl_3_:Bi crystals: coordination number, CN; interatomic distance between the central atom and the next neighbors (the length for single scattering path), *d*, Å; the Debye–Waller factor, σ^2^; the convergence factor, *R_f_*.

Sample	Scattering Path	CN	*d*_EXAFS_*,* Å	σ^2^, Å^2^	*R_f_,*%
CsCdCl_3_:Bi	Cd1–Cl1	2	2.60	0.0040	1.4
	Cd2–Cl1	2	2.56	0.0022	
	Cd2–Cl2	2	2.72	0.0019	
CsCdBr_3_	Cd–Br	6.0	2.73	0.0091	2.1
	Cd–Cd	1.8	3.38	0.0078	
CsCdBr_3_:Bi	Cd–Br	6.0	2.74	0.0094	0.8
	Cd–Cd	2.3	3.36	0.0119	

**Table 11:** The results of single-sphere EXAFS fit for the TlCdCl_3_:Bi and TlCdI_3_:Bi crystals.

Initial Charge Composition	Coordination Number (CN)		Interatomic Distance, Å
TlCdI_3_:Bi^1+^	Tl–I1	1.5	3.86
	Tl–I2	0.7	4.02
	Tl–I3	3.0	4.09
TlCdCl_3_:Bi^1+^	Tl–Cl1	2.0	2.53
	Tl–Cl2	2.0	2.73
	Tl–Cl3	2.0	3.17
TlCdI_3_:Bi^1+^	Cd–Cl1	1.3	2.58
	Cd–Cl2	4.4	2.71
TlCdCl_3_:Bi^1+^	Cd–I1	0.7	2.87
	Cd–I2	3.8	3.00
